# Divergent roles of lysyl oxidase family members in ornithine decarboxylase- and RAS-transformed mouse fibroblasts and human melanoma cells

**DOI:** 10.18632/oncotarget.26508

**Published:** 2018-12-28

**Authors:** Mari Kielosto, Johanna Eriksson, Pirjo Nummela, Miao Yin, Erkki Hölttä

**Affiliations:** ^1^ Department of Pathology, University of Helsinki, Helsinki, Finland; ^2^ Current address: University of Helsinki, Genome-Scale Biology Research Program, Helsinki, Finland

**Keywords:** lysyl oxidase family, c-Jun, ODC, RAS, melanoma

## Abstract

We have previously shown that proto-oncoprotein c-Jun is activated in ornithine decarboxylase (ODC)- and RAS-transformed mouse fibroblasts, and that the transformed morphology of these cells can be reversed by expressing the transactivation domain deletion mutant of c-Jun (TAM67). Here, we found that lysyl oxidase (*Lox*), encoding an extracellular matrix-modifying enzyme, is downregulated in a c-Jun-dependent manner in ODC-transformed fibroblasts (Odc cells). In addition to *Lox*, the Lox family members Lox-like 1 and 3 (*Loxl1 and Loxl3*) were found to be downregulated in Odc as well as in RAS-transformed fibroblasts (E4), whereas Lox-like 4 (*Loxl4*) was upregulated in Odc and downregulated in E4 cells compared to normal N1 fibroblasts. Tetracycline-regulatable LOX re-expression in Odc cells led to inhibition of cell growth and invasion in three-dimensional Matrigel in an activity-independent manner. On the contrary, *LOX* and especially *LOXL2*, *LOXL3*, and *LOXL4* were found to be upregulated in several human melanoma cell lines, and LOX inhibitor B-aminopropionitrile inhibited the invasive growth of these cells particularly when co-cultured with fibroblasts in Matrigel. Knocking down the expression of LOX and especially LOXL2 in melanoma cells almost completely abrogated the invasive growth capability. Further, *LOXL2* was significantly upregulated in clinical human primary melanomas compared to benign nevi, and high expression of *LOXL2* in primary melanomas was associated with formation of metastases and shorter survival of patients. Thus, our studies reveal that inactive pro-LOX (together with Lox propeptide) functions as a tumor suppressor in ODC- and RAS-transformed murine fibroblasts by inhibiting cell growth and invasion, and active LOX and LOXL2 as tumor promoters in human melanoma cells by promoting their invasive growth.

## INTRODUCTION

Proto-oncoprotein c-Jun is a transcription factor, a member of the AP-1 (activation protein-1) transcription factor family. It can dimerize through its leucine zipper motif with other members of the AP-1 family (reviewed in [1]). c-Jun is involved in many cellular activities, such as cell proliferation [2, 3], apoptosis [4], differentiation [5, 6], tumor invasion [7, 8], metastasis [9], and angiogenesis [10, 11]. c-Jun expression and activity have been found to be elevated in cell lines transformed by many different oncoproteins, such as receptor tyrosine kinases, Src, Ras, Raf, Fos, and Myc [12, 13], and in human cancers, such as pancreatic cancer [14], breast cancer [15], sarcomas [16], glioblastoma [17], and melanoma [18, 19].

Ornithine decarboxylase (ODC) is a key regulatory enzyme in the biosynthesis of polyamines. Like c-Jun, also ODC is essential for mammalian development and tumor formation [20]. In our earlier studies, we have shown that the overexpression of human ODC induces morphological transformation of immortalized NIH3T3 cells [21] and gives rise to rapidly growing fibrosarcomas in *nude* mice [22]. ODC-induced transformation was associated with constitutive c-Jun activation [23], and induced expression of the transactivation domain deletion mutant of c-Jun (TAM67) was found to reverse the transformed morphology and reduce their invasive growth [24]. Similar results were obtained with RAS-transformed mouse fibroblasts (E4 cells) [24].

Lysyl oxidase (LOX) is a secreted copper-dependent amine oxidase that plays an important role especially in the crosslinking of collagen and elastin in the extracellular matrix [25]. LOX is synthesized and secreted as a 50-kDa inactive glycosylated proenzyme (pro-LOX), which is then cleaved extracellularly into a functional 32-kDa enzyme (LOX) and an 18-kDa propeptide (LOX-PP) by bone morphogenetic protein 1 (BMP-1) and related proteases (Tolloid-like 1 and 2) [26]. LOX-PP can further exist in differentially glycosylated forms of higher molecular weight up to 35 kDa [27]. LOX has been reported to control cell phenotype and regulate many cellular processes, including cell adhesion, migration, and invasion [28–31], as well as epithelial-mesenchymal transition in hypoxic conditions [32, 33]. Paradoxically, LOX has been reported to function both as a tumor suppressor and a promoter in human cancer cells, depending on tumor type and stage of progression. Originally, *Lox* (first named the *ras recision gene, rrg*) was proposed to be a tumor suppressor gene when it was found to suppress HRAS-induced transformation in mouse cells [34, 35]. LOX has also been found to function as a tumor suppressor in basal and squamous cell carcinoma, gastric cancer, and osteosarcoma cells [36–38], but as a tumor promoter in colorectal cancer, esophageal squamous cell carcinoma, lung adenocarcinoma, and laryngeal cancer [39–42].

In addition to LOX, four other LOX family members designated as LOX-like proteins LOXL1-LOXL4 have been identified in mammals. LOX and LOXL proteins contain a conserved C-terminal domain, required for the amine oxidase activity, and differing N-terminal sequences, which may confer individual functions [43]. LOX and LOXL proteins are expressed in different developmental states and in different tissues, and they seem to be unable to functionally compensate each other. Like LOX, also LOX-like proteins may function as tumor suppressors [44] or promoters [45–47].

In the present study, we first sought to identify c-Jun- and transformation- specific genes downregulated in the ODC-transformed fibroblasts (Odc cells) by genome-wide microarray analysis. We found that one of the genes downregulated by c-Jun in Odc cells is *Lox*. We further studied the significance of it in the ODC-induced transformation by generating cell lines with inducible LOX (pro-LOX) expression. In addition to *Lox*, expression levels of the other Lox family members, *Lox-like-1-4*, were also studied in these cells and in the RAS-transformed (E4) cells. As we had earlier incidentally noticed by microarray analysis two melanoma cell lines to overexpress *LOXL2* [48], we additionally studied the expression levels of all LOX family genes in different melanoma cell lines. In contrast to that in ODC-transformed fibroblasts, we found a general increase in the expression of the LOX family members in melanoma cells. To resolve this paradox, we further studied the functions of the encoded proteins by using a universal LOX inhibitor Β-aminopropionitrile (BAPN) and knocking down of LOX and LOXL2 in melanoma cells. Our data suggest that inactive pro-LOX functions as a tumor suppressor in ODC- and RAS-transformed mouse fibroblasts by inhibiting cell growth and invasion, and that the mature, active LOX and LOXL2 act as tumor promoters in human melanoma cells by promoting their invasive growth. Further, we show that high LOXL2 mRNA expression may be correlated with metastasis and poor survival in melanoma.

## RESULTS

### LOX expression is downregulated in ODC-transformed mouse fibroblasts in a c-Jun-regulated manner

**Figure 1:**
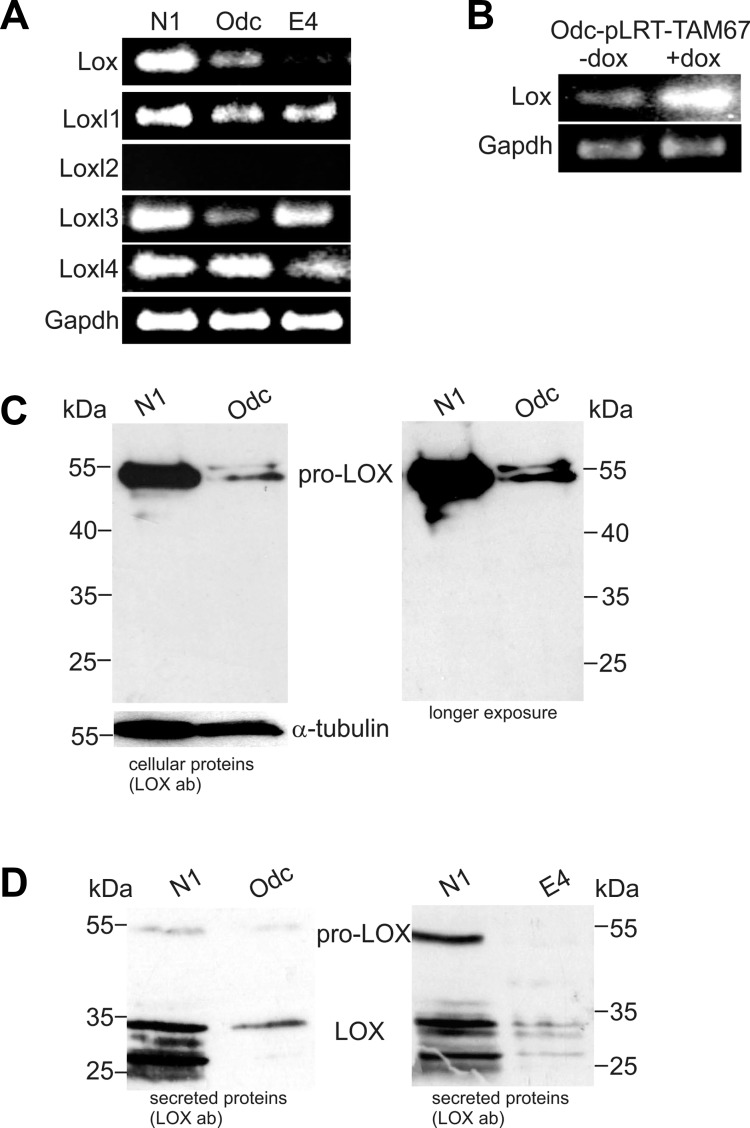

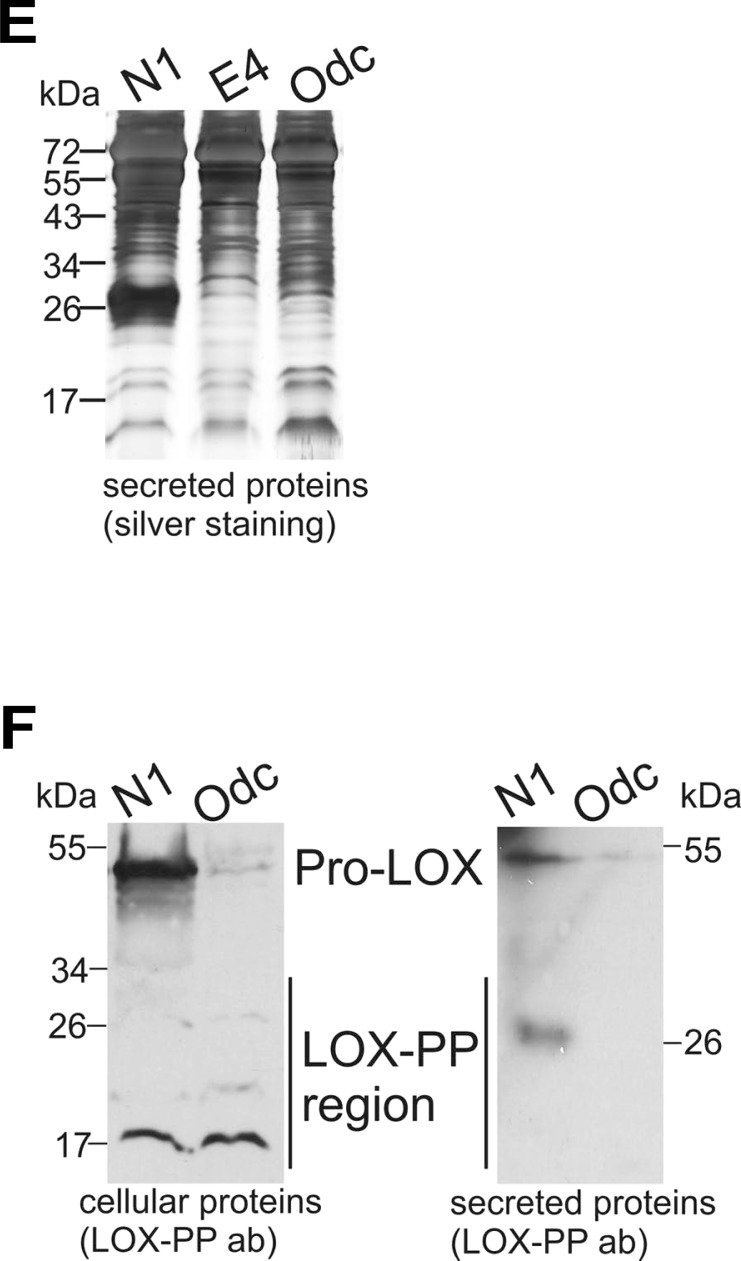
Expression levels of Lox and Lox-like1-4 in normal (N1), ODC-transformed (Odc), and Ras-transformed (E4) mouse fibroblasts (**A**) Lox and Loxl1-4 mRNA levels in N1, Odc, and E4 cell lines analyzed by RT-PCR. (**B**) RT-PCR analysis of Lox mRNA expression levels in ODC-transformed fibroblasts transfected with inducible pLRT-TAM67 vector (Odc-pLRT-TAM67 cells) in the absence (−dox) and presence (+dox) of 1 μg/ml doxycycline. Gapdh was used as a control. (**C**) Western blot analysis of cellular (40 μg protein/lane) pro-Lox and Lox protein levels in N1 and Odc cell lines and (**D**) secreted Lox protein levels in N1, Odc, and E4 cell lines (17 μg protein/lane). (**E**) Silver staining of total secreted proteins in samples used in (D). (**F**) Western blot analysis of cellular (40 μg protein/lane) and secreted (17 μg protein/lane) pro-Lox and LOX-PP proteins in N1 and Odc cells detected with a LOX propeptide-specific antibody.

In this study, we first set out to identify ODC-induced transformation-associated genes downregulated by c-Jun. By using gene expression microarray analyses, we searched for genes that are both downregulated in ODC-transformed cells (Odc cells) compared to parental N1 fibroblasts as well as upregulated in Odc cells transfected with a tetracycline-inducible TAM67 vector (Odc-pLRT-TAM67) after induction of TAM67 expression. Using two different microarray platforms, only three genes - fibulin 5 (*Fbln5*), *Lox*, and microfibrillar associated protein 5 (*Mfap5*) (all encoding extracellular matrix proteins) - showed ≥ 2-fold changes in their expression levels in the two comparisons (Table 1). As *Lox* has been proposed to be a tumor suppressor and also to be downregulated in HRAS-transformed mouse cells [34, 35], we selected it to be studied in more detail. First, we verified by RT-PCR the downregulation of *Lox* in Odc cells, and the upregulation of *Lox* in Odc-pLRT-TAM67 cells, after TAM67 induction (Figure 1A and 1B). We further studied the expression of *Lox* in the RAS-transformed (E4) cells and found its expression to be downregulated compared to N1 cells (Figure 1A), consistent with previous findings [34, 35]. The downregulation of *Lox* expression in Odc cells was also seen at the protein level. Immunoblotting with a LOX antibody recognizing both pro-LOX and mature LOX revealed that the normal N1 cells contained high levels of pro-LOX but no detectable amounts of cleaved/mature LOX, and that the transformed Odc cells showed a marked decrease in pro-LOX expression (Figure 1C). Analysis of the secreted proteins from the cells with the same antibody showed that pro-LOX was secreted and cleaved to mature/active LOX, roughly proportionally to the cellular levels of pro-LOX (Figure 1D and 1E). The cellular protein levels of the LOX-propeptide region, detected by LOX-PP antibody (Figure 1F, left panel), showed no clear difference between the N1 and Odc cells. However, when analyzing the secreted proteins, a 26 kDa protein band was detected in N1 cells, but not in Odc cells (Figure 1F, right panel). The 26 kDa band may well represent glycosylated LOX propeptide [49]. The 18 kDa protein band seen in the cell extracts equally expressed in the N1 and Odc cells (Figure 1F, left panel) is unlikely to be LOX-PP, but represents a protein non-specifically binding the antibody in NIH3T3 cells [50].

**Table 1:** Identification of genes downregulated in ODC-transformed NIH3T3 cells (Odc) compared to parental N1 cells and upregulated in Odc cells expressing a tetracycline-inducible TAM67 vector (Odc-pLRT-TAM67) by Incyte genomics cDNA and Affymetrix oligonucleotide microarrays

Genebank	Gene	Incyte		Affymetrix	
		Odc vs N1	Odc-pLRT-TAM67 +dox vs −dox	Odc vs N1	Odc-pLRT-TAM67 +dox vs −dox
AA437518	Fibulin 5 (*Fbln5*)	−5.5	4.4	−8.8	3
W83882	Lysyl oxidase (*Lox*)	−3.3	2.5	−3.4	2.2
AA037995	Microfibrillar associated protein 5 (*Mfap5*)	−24.2	2.1	−14	2

Values are fold changes.

+dox = with 1 μg/ml doxycycline for 3 days.

−dox = without doxycycline for 3 days.

### Lox and Lox-like 1 and 3 are downregulated in Odc and E4 cells

In addition to *Lox*, we also studied the expression of other Lox family members, *Lox-like 1-4*, in parental N1 fibroblasts and in ODC- and RAS-transformed (E4) cells by Affymetrix microarrays (Table 2). Of the Lox family members, the highest expression levels in the cells were seen with *Lox*. Besides *Lox*, we found *Loxl1*, and *Loxl3* to be downregulated both in Odc and E4 cells compared to normal N1 cells (Table 2). *Loxl4* in turn was slightly upregulated in Odc cells but downregulated in E4 cells. The expression levels of *Lox*, *Loxl1*, *Loxl3*, and *Loxl4* were further verified by RT-PCR (Figure 1A). *Loxl2* was not expressed to any appreciable extent in any of these cell lines (Figure 1A), in agreement with the microarray data (Table 2).

**Table 2:** The gene expression levels of Lox family members in normal (N1), ODC-transformed (Odc), and Ras-transformed (E4) NIH 3T3 cells analyzed by Affymetrix MOE430 set arrays

Probeset Id	Gene	Signal			Fold	
		N1	Odc	E4	Odc vs N1	E4 vs N1
1416121_at	Lysyl oxidase (*Lox*)	7849	2223	1268	−3.6	−6.3
1448228_at	Lysyl oxidase (*Lox*)	7385	2167	1070	−3.4	−7.1
1451978_at	Lysyl oxidase-like 1 (*Loxl1*)	2270	696	575	−3.2	−4.0
1418269_at	Lysyl oxidase-like 3 (*Loxl3*)	1033	207	399	−5.0	−2.6
1421153_at	Lysyl oxidase-like 4 (*Loxl4*)	311	539	157	1.7	−2.0
1450134_at	Lysyl oxidase-like 4 (*Loxl4*)	320	481	156	1.5	−3.1

Note: lysyl oxidase-like 2 (*Loxl2*) expression was absent in all the cell lines.

Noteworthy, we also found ODC-transformed cells to downregulate, in addition to *Lox*, the expression of many other extracellular matrix (ECM)-related genes compared to the parental NIH3T3 fibroblasts, including *Col1a1* 4.1-fold, *Col1a2* 2.0-fold, and *Fn1* 10.6-fold. Of the downregulated genes 7.4% (56/759) encoded ECM proteins, whereas only 0.7% (7/1076) of the upregulated genes were ECM-related (data not shown).

### Re-expression of pro-LOX inhibits the growth and invasion of Odc cells

**Figure 2:**
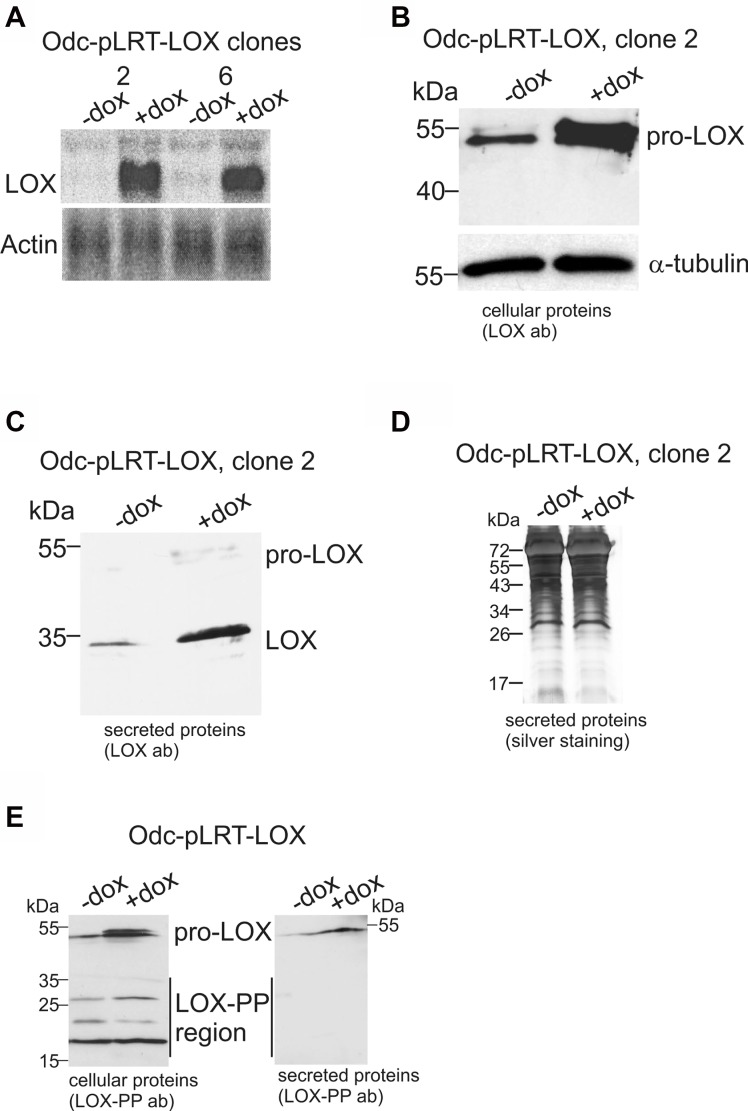
Ectopic expression of LOX in Odc cells by using tetracycline-inducible system (**A** and **B**) LOX mRNA and protein expression levels in the Odc-pLRT-LOX clones in the absence (−dox) and presence (+dox) of doxycycline, as analyzed by Northern blotting (10 μg RNA/lane) (A) and Western blotting (40 μg protein/lane) (B). Actin was used as a loading control in Northern blotting (A) and α-tubulin in Western blotting (B). (**C**) Analysis of the secreted LOX protein levels (17 μg protein/lane) as analyzed by Western blotting. (**D**) Silver staining of total secreted proteins in samples used in (C). (**E**) Western blot analysis of cellular (40 μg protein/lane) and secreted (17 μg protein/lane) pro-LOX and LOX-PP proteins with LOX-PP antibody in Odc-pLRT-LOX cells, without or with induction of lysyl oxidase expression.

To study the functional significance of LOX, we generated cell lines carrying a tetracycline-inducible expression system of LOX cDNA (pro-LOX) in ODC-transformed cells (Odc-pLRT-LOX). Cell clones with a robust LOX mRNA expression by doxycycline induction and low background expression in the absence of doxycycline (Figure 2A) were then selected for more detailed studies. The induction of LOX expression was also confirmed at the protein level (Figure 2B). The induced pro-LOX was further verified to be secreted and correctly processed to mature LOX by analysis of the secreted proteins (Figure 2C). Silver staining showed that doxycycline did not affect total protein levels (Figure 2D). Analysis of the cellular proteins with the LOX propeptide antibody showed an increase in pro-LOX expression upon doxycycline induction, but no clear difference could be detected in the protein levels of the LOX-PP region between the non-induced and induced states (Figure 2E, left panel). Immunoblotting of the secreted proteins with the LOX-PP antibody revealed also an increase in pro-LOX, but not in LOX-PP, upon induction of the cells with doxycycline (Figure 2E, right panel). Apparently, the amount of LOX-PP among the secreted proteins was too low to be detected with the antibody.

**Figure 3:**
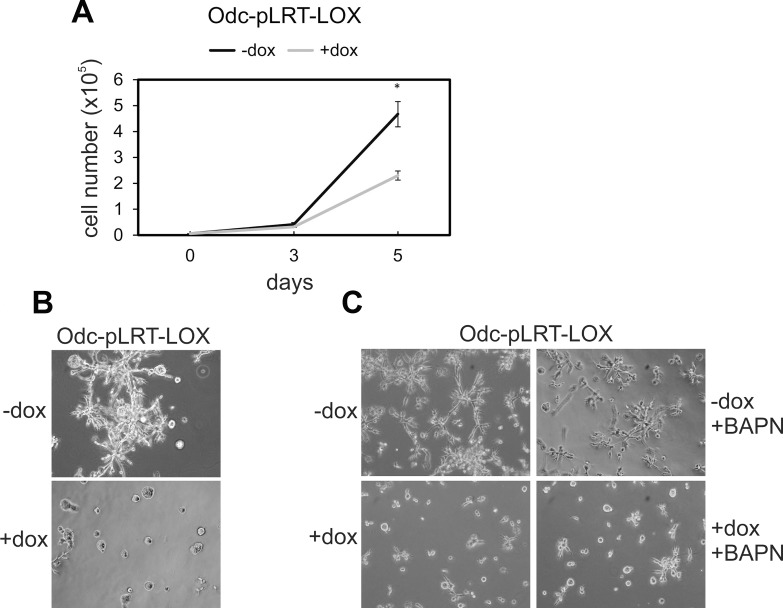
Effect of ectopic LOX expression on cell growth and invasion of ODC-transformed fibroblasts transfected with a tetracycline-inducible expression system (Odc-pLRT-LOX) (**A**) Growth curves of Odc-pLRT-LOX cells grown for 5 days in the absence (−dox) or presence (+dox) of doxycycline. Data are expressed as means ± SD of three parallel cultures. ^*^p = 0.0014 at 5 days of culture. (**B**) Odc-pLRT-LOX cells grown between two thick layers of Matrigel in the absence or presence of doxycycline and photographed after 3 days. (**C**) Odc-pLRT-LOX cells grown in Matrigel without (−dox and −dox+BAPN) or with (+dox and +dox+BAPN) doxycycline and with LOX inhibitor ΒAPN (500 μM) (−dox+BAPN and +dox+BAPN) and photographed after 2 days.

Then, we tested the effect of pro-LOX expression on the growth rate of Odc cells and found the induced expression of pro-LOX to significantly inhibit (*p* = 0.0014) the growth of Odc-pLRT-LOX cells (Figure 3A). Empty vector-bearing cells showed no change in growth after doxycycline induction (data not shown).

To test whether Lox is also involved in the regulation of cell invasion, we used a 3D-Matrigel assay mimicking the situation *in vivo*. The induced expression of pro-LOX resulted in a marked inhibition of the invasive growth of Odc-pLRT-LOX cells (Figure 3B). Doxycycline had no effect on the invasive growth pattern of Odc cells with empty control vector (data not shown). Next, to study whether the extracellular processing of pro-LOX to active LOX enzyme/ the enzyme activity had any role in the inhibition of invasion, we incubated the cells with BAPN, a competitive, irreversible, active-site directed inhibitor of LOX [51], in the absence or presence of doxycycline. Although the IC_50_ value of BAPN for different LOX substrates is in the micromolar range (1–15 μM) [51–53], higher concentrations of 250 to 500 μM BAPN were required to fully block the LOX activity in the conditioned medium of the cells (Supplementary Figure 1A), consistent with previous findings on other cell lines [52, 53]. At these concentrations, BAPN is still known to be sufficiently specific to LOX [54]. Further, the other possible BAPN-targetable amine oxidases, like AOC1 (diamine oxidase) and AOC3 (VAP-1/SSAO), are not appreciably expressed in the cell lines studied here (data not shown), adding to the specificity of BAPN action. The inhibitor at a 500 μM concentration did not either show any toxicity, as evidenced by no interference with the proliferation of Odc cells in 2D cell culture (Supplementary Figure 2) or the invasive activity of the cells in 3D-Matrigel (Figure 3C). Thus, the doxycycline-induced expression of pro-LOX blocked the invasive capability of the cells irrespective of LOX activity.

### LOX family members are variably expressed in different human melanoma cell lines

**Figure 4:**
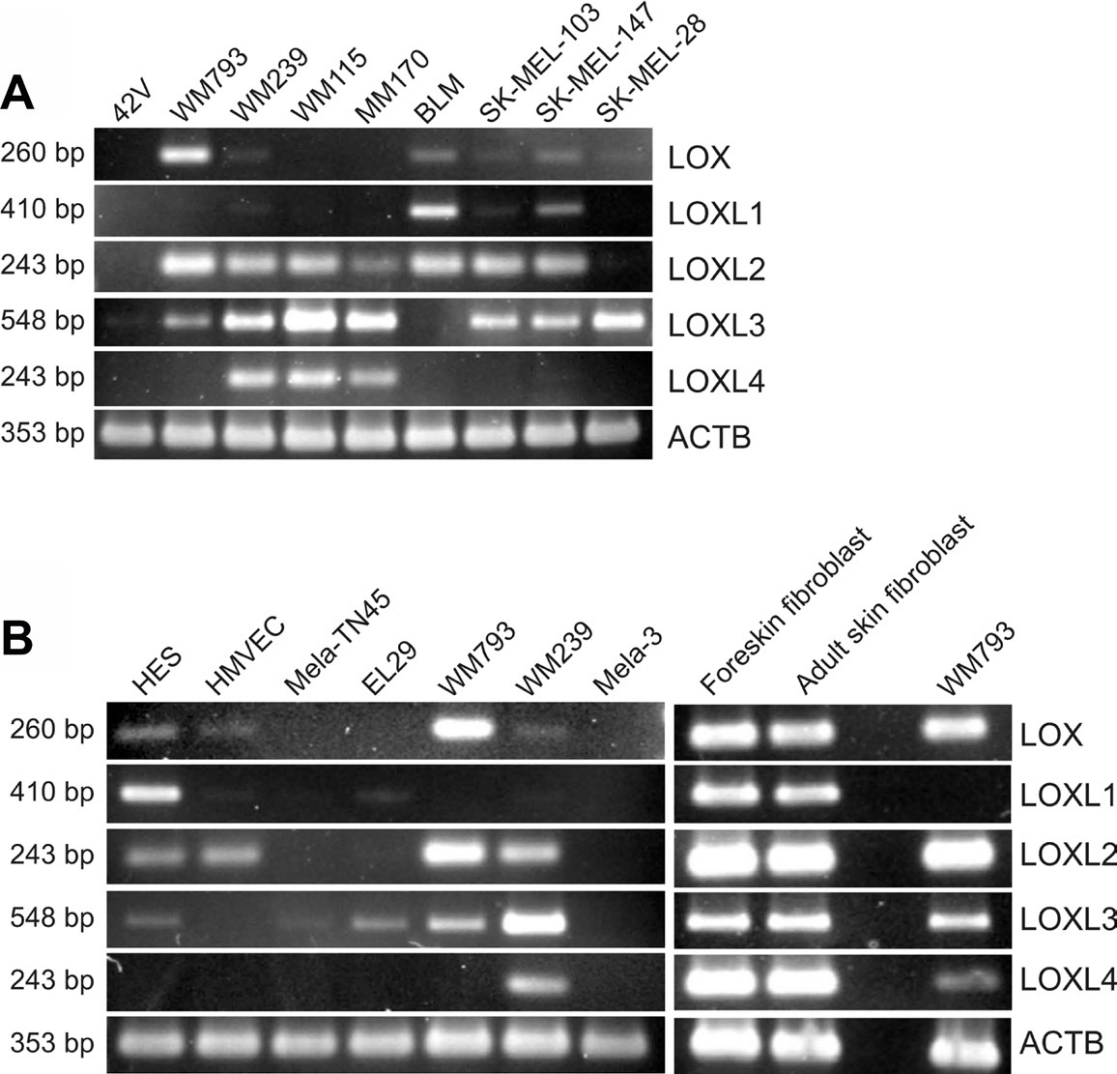
Expression of LOX family members in different human normal cells, primary melanoma cells, and melanoma cell lines (**A**) Expression of LOX and LOXL1-4 mRNAs in normal melanocytes (42V) and a panel of melanoma cell lines, and (**B**) in human embryonic fibroblasts (HES), human microvascular endothelial cells (HMVECs), normal melanocytes (Mela-TN45 and Mela3), primary melanoma cells (EL29), and in WM793 and WM239 melanoma cell lines, as well as in primary fibroblasts, as analyzed by RT-PCR. Beta-actin (ACTB) was used as a loading control.

**Figure 5:**
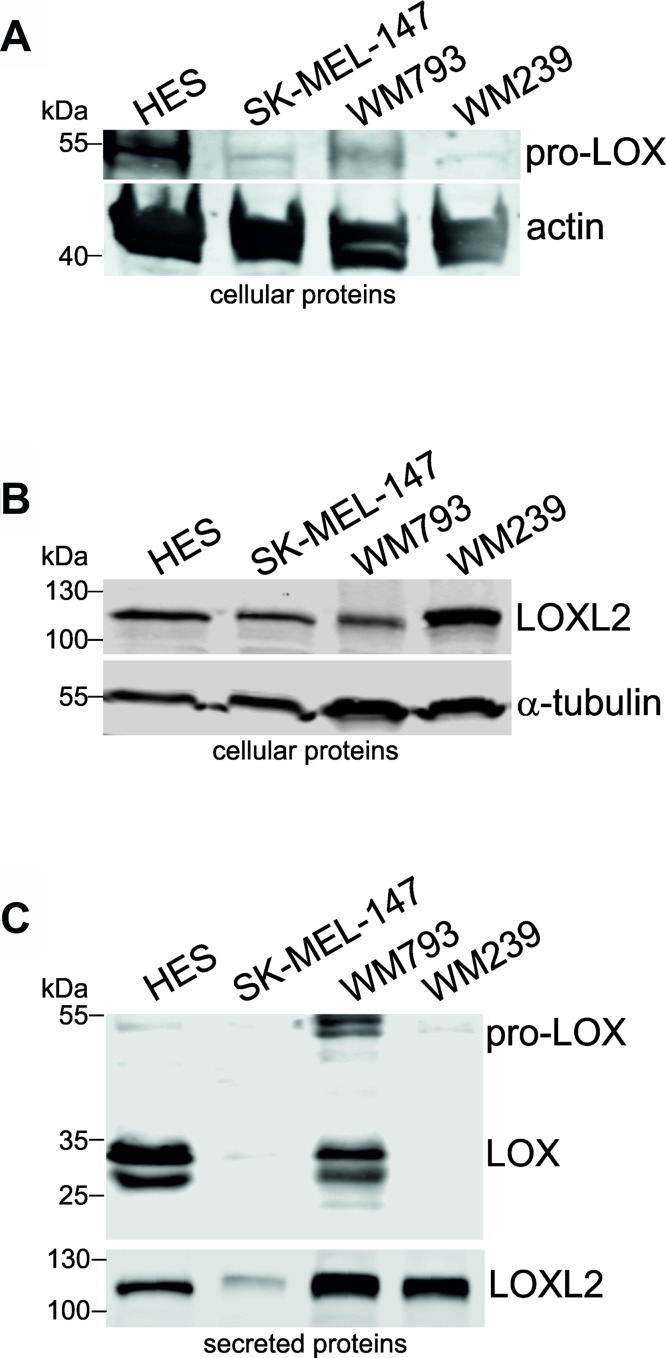
Protein expression levels of LOX and LOXL2 in human fibroblasts and melanoma cell lines (**A** and **B**) Western blot analysis of cellular pro-LOX (A) and LOXL2 (B) protein levels in human embryonic fibroblasts (HES), and melanoma cell lines SK-MEL-147, WM793, and WM239. Actin and alpha-tubulin were used as the loading controls. (**C**) Western blot analysis of secreted LOX and LOXL2 protein levels in HES cells, and melanoma cell lines SK-MEL-147, WM793, and WM239.

In addition to that in fibroblast transformation, we have incidentally noticed in our microarray analyses of two melanoma cell lines that the expression levels of LOX family members change during the development of human melanoma cells [48]. As *LOXL2* was one of the most highly upregulated genes in melanoma cells compared to normal melanocytes [48], we were interested to investigate the role of all LOX family members in melanoma cells. First, we studied the mRNA expression levels of *LOX* and *LOXL1-4* in a panel of melanoma cell lines and primary melanoma cells, as well as in primary human melanocytes, fibroblasts, and endothelial cells. Interestingly, *LOXL2* and *LOXL3* were found to be expressed in nearly all the melanoma cell lines studied (Figure 4A). *LOX* was expressed at the level of assay sensitivity only in the vertical growth phase melanoma cell line WM793 and in the metastatic cell lines BLM and SK-MEL-147. *LOXL1*, in turn, was expressed only in BLM and SK-MEL-147 cell lines, and *LOXL4* in the vertical growth phase cell line WM115 and in the metastatic cell lines WM239 and MM170. The primary melanocytes 42V, Mela-3, and Mela-TN45 did not express any of the LOX family members (Figure 4A and 4B). Human embryonic skin fibroblasts (HES), in turn, expressed nearly all the mRNAs (except *LOXL4*), while the endothelial cells (HMVECs) expressed only *LOXL2* (Figure 4B). Two non-embryonic cultures of human primary fibroblasts expressed all LOX family members at high levels (Figure 4B). The cellular and secreted protein levels of LOX and LOXL2 in fibroblasts (HES) and melanoma cells (SK-MEL-147, WM793 and WM239) were then studied (Figure 5). Pro-LOX exhibited high expression in HES and lower variable expression in melanoma cells, with WM793 showing the highest expression among them (Figure 5A). The protein expression of LOXL2, in turn, was abundant in both HES and the melanoma cells, and varied less than that of LOX, with SK-MEL-147 showing the lowest expression (Figure 5B). Analysis of the secreted proteins showed that pro-LOX was efficiently processed to the active LOX, particularly in HES cells, while LOXL2 remained unprocessed (Figure 5C).

**Figure 6:**
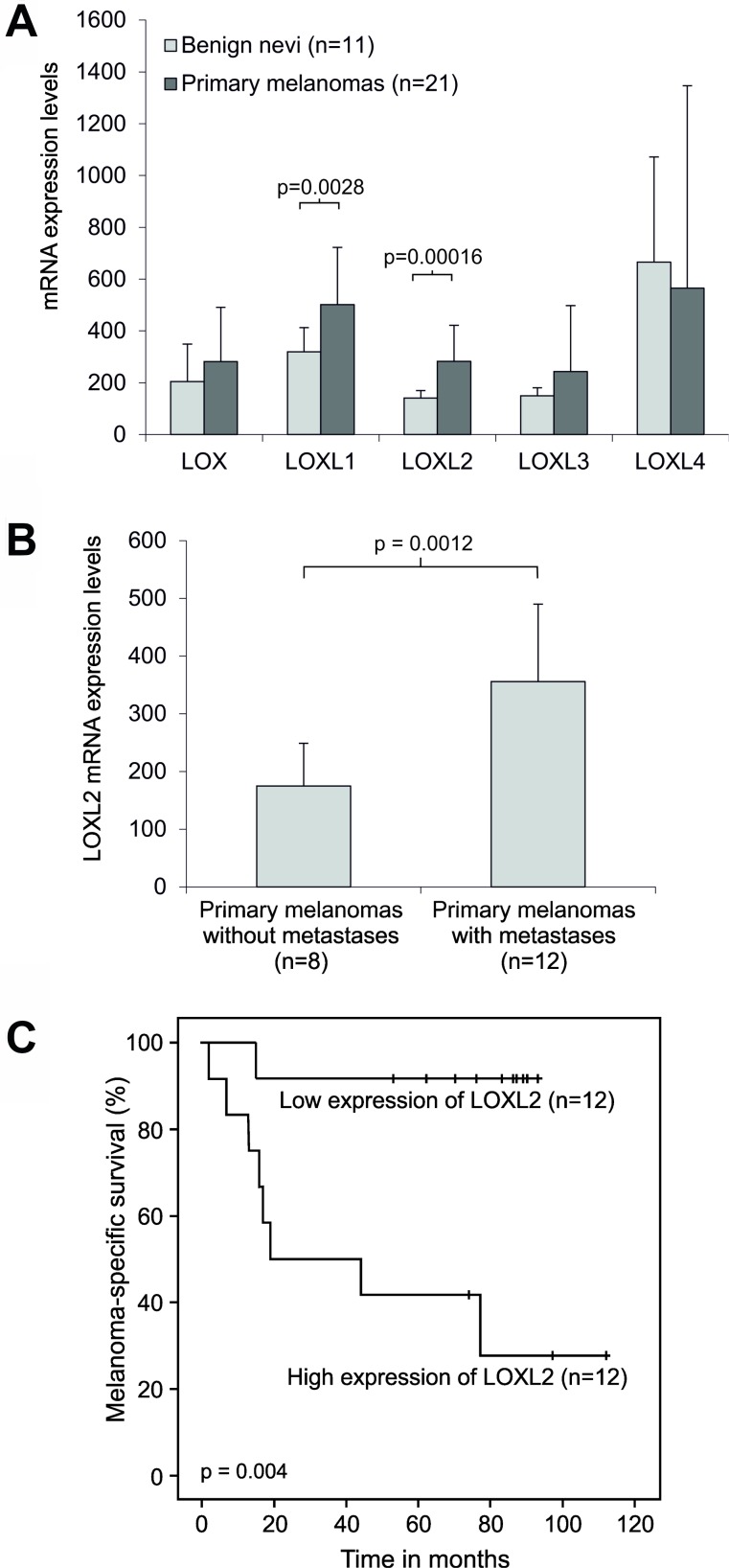
Expression of LOX and LOX-like genes in clinical human melanoma samples (**A**) Comparison of the mean mRNA expression levels of LOX and LOX-like genes in benign nevi and primary melanomas. (**B**) Comparison of the mean mRNA expression levels of LOXL2 in primary melanomas with and without metastases. The relative mRNA levels were measured by Affymetrix HG U133 Plus 2.0 arrays. Error bars indicate SDs. (**C**) Kaplan–Meier survival curves for melanoma patient groups with primary melanoma showing low and high LOXL2 mRNA expression measured by Affymetrix HG U133 Plus 2.0 and HG U133 set arrays.

In addition to cell lines, we also compared the mRNA expression levels of LOX and LOXL1-4 in clinical tissue specimens of human benign nevi and malignant melanomas, and found *LOXL1* (fold 1.6; *p* = 0.0028) and *LOXL2* (fold 2.0; *p* = 0.00016) to be significantly upregulated in the primary melanomas (Figure 6A). In addition, LOXL2 mRNA expression levels were significantly upregulated (fold 2.0; *p* = 0.0012) in the metastatic compared to non-metastatic primary melanomas (Figure 6B). Further, we compared the Kaplan–Meier survival curves of patients with primary melanomas displaying low and high expression of *LOXL2* and found a significant association between high expression of *LOXL2* and shorter survival time (Figure 6C).

### Inhibition of LOX activity suppresses the invasive growth of human melanoma cells and fibroblasts

**Figure 7:**
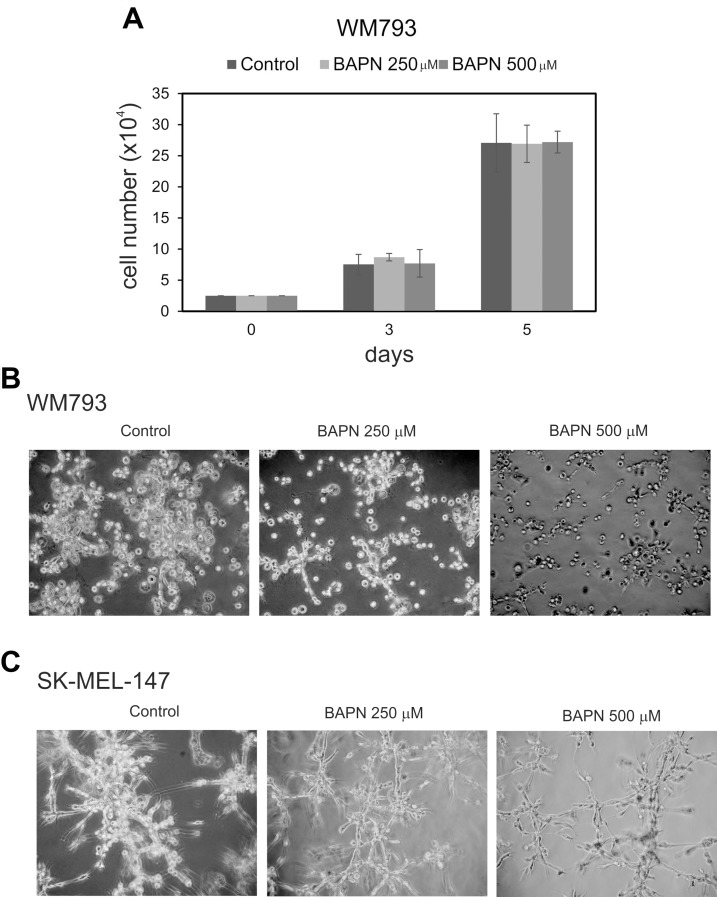
Effects of LOX inhibitor β-aminopropionitrile (BAPN) on the proliferation and invasive growth of melanoma cells (**A**) The cell numbers of WM793 melanoma cells grown for 3 and 5 days in the presence of 0, 250, or 500 μM ΒAPN. 10^4^ cells were initially plated. (**B** and **C**) WM793 (B) and SK-MEL-147 (C) melanoma cells cultured in 3D Matrigel without or with BAPN at 250 or 500 μM concentrations; 3 × 10^4^ WM793 and 2 × 10^4^ SK-MEL-147 melanoma cells were plated between the two layers of Matrigel; WM793 was cultured for 4 days and SK-MEL-147 for 3 days.

**Figure 8:**
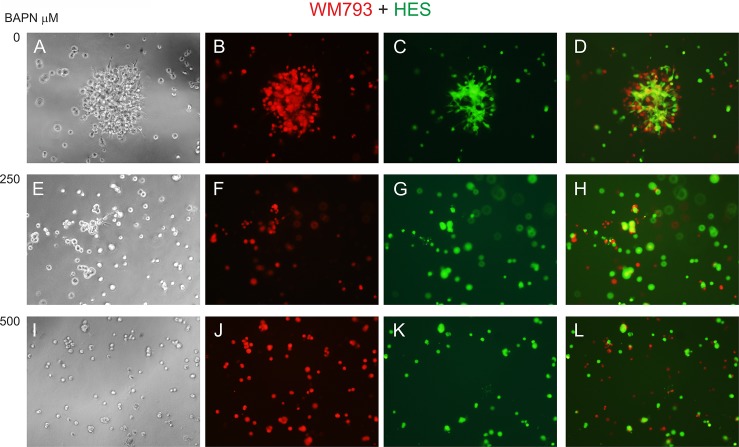
Effect of LOX inhibitor BAPN on the invasive growth of co-cultured WM793 melanoma cells and human embryonic fibroblasts (HES) Fluorescent-labeled WM793 cells (10^4^) (red) (B, F, J) and fibroblasts (10^4^) (green) (C, G, K) co-cultured in 3D Matrigel without (**A**–**D**) and with BAPN at 250 (**E**–**H**) or 500 (**I**–**L**) μM concentrations for 24 hours. D: merged image of B and C; H: merged image of F and G; L: merged image of J and K.

**Figure 9:**
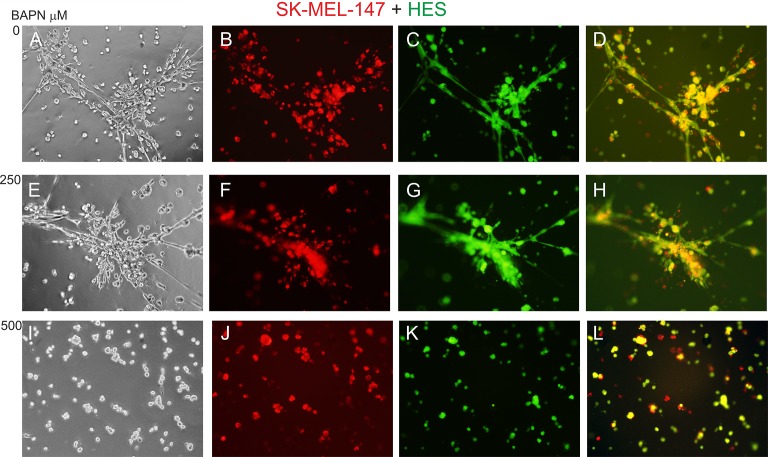
Effect of LOX inhibitor BAPN on the invasive growth of co-cultured SK-MEL-147 melanoma cells and HES cells Fluorescent-labeled SK-MEL-147 (red) (B, F, J) and fibroblasts (green) (C, G, K) co-cultured (1:1) in 3D Matrigel without (**A**–**D**) or with BAPN at 250 (**E**–**H**) or 500 (**I**–**L**) μM concentrations for 24 hours. D: merged image of B and C; H: merged image of F and G; L: merged image of J and K.

To study the effects of LOX family members on melanoma cell behavior, we first inhibited LOX activity in WM793 cells with 250 and 500 μM BAPN, which effectively blocked the enzyme activity *in vitro* (Supplementary Figure 1B), and studied cell proliferation in standard 2D culture. BAPN at these concentrations did not interfere with cell growth, when cultured at ordinary cell densities (Figure 7A). However, a minor, non-significant (*p* = 0.12) decrease in the proliferation rate was detected in sparse cell cultures (Supplementary Figure 3). We further checked that BAPN did not induce apoptosis in WM793 and SK-MEL-147 cells at these concentrations (data not shown). By contrast, BAPN (500 μM) completely abrogated the low invasive activity of WM793 cells, and partially inhibited the high invasive capability of SK-MEL-147 cells in 3D Matrigel (Figure 7B and 7C). Notably, when the melanoma cells were co-cultured in 3D Matrigel with fibroblasts (which we have found to dramatically promote the invasion of melanoma cells [55]), inhibition of LOX activity led to a complete inhibition of the co-invasive growth of fibroblasts and melanoma cells (both WM793 and SK-MEL-147 cells; WM793 already with 250 μM BAPN) (Figures 8 and 9). We further used fluorescent labeling of melanoma cells (red) and fibroblasts (green) to differentiate the effect of LOX inhibition on the two cell types in the co-cultures (Figures 8 and 9). In the absence of the inhibitor, fibroblasts became spindle shaped/activated and came into direct contact with the melanoma cells, as we have previously reported [55]. Inhibition of LOX activity blocked the activation of fibroblasts (cells remained round) and inhibited the subsequent growth and invasion of both cell types (Figures 8 and 9).

### Knockdown of LOX and LOXL2 inhibit the proliferation and invasive growth of WM793 melanoma cells

**Figure 10:**
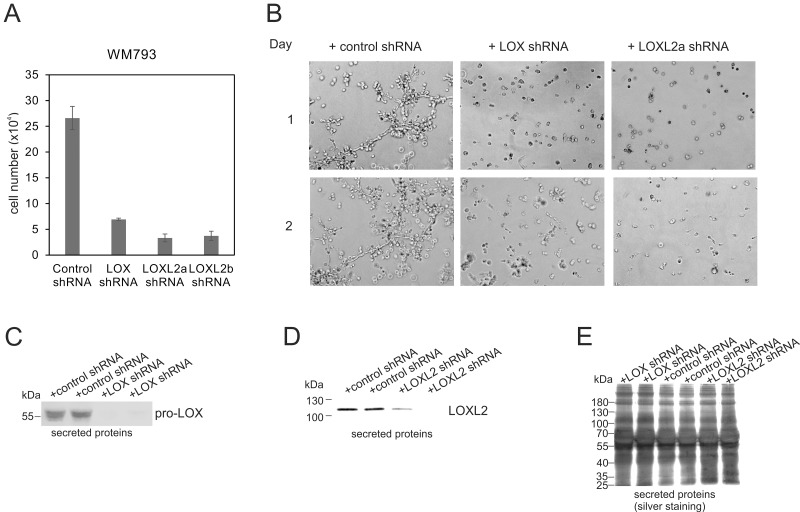
Effects of LOX- and LOXL2- knockdowns on the proliferation and invasive growth of WM793 melanoma cells (**A**) The proliferation of WM793 melanoma cells 10 days after the lentiviral transduction of control, LOX, LOXL2a (clone TRCN0000046195) and LOXL2b (clone TRCN0000046197) shRNAs. Data are expressed as means ± SD of three parallel cultures. *p* < 0.001 in all (control shRNA versus LOX, LOXL2a or LOXL2b shRNAs). (**B**) WM793 melanoma cells cultured in 3D Matrigel 3 weeks after the transduction with lentiviral control, LOX, or LOXL2a (clone TRCN0000046195) shRNAs. The cells were photographed after 1 and 2 days. (**C** and **D**) Western blot analysis of LOX (C) and LOXL2 (D) protein levels in the conditioned media of WM793 cells. The cells transduced in duplicates with lentiviral control and LOX shRNAs (C) and control and LOXL2 (clone TRCN0000046195) shRNAs (D) were passaged for 32 days in normal serum containing media and changed to serum free medium for 18 hours before collecting the conditioned media. (**E**) Silver staining of total secreted proteins in samples used in (C and D).

Finally, we examined the functional consequences of the depletion of LOX and LOXL2 proteins by specific short-hairpin RNAs (shRNAs) in WM793 cells. We found that the proliferative capacity of the cells in standard 2D culture was markedly reduced (Figure 10A), and that the invasive growth capacity in 3D Matrigel was almost totally inhibited (Figure 10B) after knocking down LOX (Figure 10C). The depletion of LOXL2 with two different shRNAs (TRCN0000046195 and TRCN0000046197) (Figure 10D and 10E) resulted in even more extensive reduction in cell proliferation than that of LOX in 2D culture (Figure 10A), and the invasive growth capability of the cells in 3D Matrigel was completely blocked during the follow-up for three days (Figure 10B and data not shown). As the antiproliferative effect of the knockdowns results in counter selection of the high shRNA expressing cells during an extended culture, inducible shRNA expression models should preferably be developed for future studies.

## DISCUSSION

LOX family members have been proposed to play multiple, complex roles in cancer, functioning both as tumor suppressors and metastasis promoters. Here, we show *Lox* to be downregulated in ODC-transformed mouse NIH3T3 fibroblasts in a c-Jun-dependent manner and to inhibit the proliferation and invasive growth of these cells. Also other members of the Lox gene family, *Loxl1* and *Loxl3*, were downregulated in Odc cells. We have previously demonstrated that ODC is a downstream effector of mutant HRAS-induced transformation in NIH3T3 cells [56, 57]. In cells that have been transformed by the *RAS* oncogene or genes signaling through RAS, the tumor suppressive action of LOX has mostly been linked to LOX-PP, which is extracellularly cleaved from pro-LOX and then uptaken by the cells [49, 58–61]. In these studies, LOX-PP has been found to reduce cell proliferation and migration *in vitro* and tumor formation *in vivo*, through inhibiting Akt, MAPK, and NFκB activation. In addition, LOX-PP has been reported to have extracellular sites of action [62, 63]. In our study, we could not detect LOX-PP in the cellular extracts of normal NIH3T3 cells (N1), which contained only pro-LOX, consistent with a previous study by Contente *et al*. [50]. In the analysis of secreted proteins from N1 cells, we detected pro-LOX, mature LOX, and LOX-PP (a 26-kDa band, apparently representing glycosylated LOX-PP), in line with the known secretion and cleavage of pro-LOX [26]. Further, in an inducible LOX expression model in Odc cells, we did not either detect any appreciable increases in LOX-PP in the cellular extracts, nor in the secreted proteins. Apparently the secreted levels of LOX-PP were too low to be detected immunologically with the current system. However, the transformed phenotype of the cells was reverted to normal upon induction of LOX expression. The morphological reversion was further found to occur in the presence of the inhibitor of LOX activity, BAPN. All these data together suggest that under physiological conditions pro-LOX, and not LOX-PP, is the main tumor suppressor in NIH3T3 cells. This conclusion is also supported by the findings of Contente *et al*. [50] on normal and HRAS-transformed NIH3T3 cells. However, the latter study did not include analyses of secreted proteins, and the authors also appear to have expressed LOX cDNA without the signal sequence, in which case the protein is not normally secreted. It is clear, however, that also LOX-PP has tumor suppressive functions, and several lines of evidence show that LOX-PP may be a good pharmacological agent to be used in cancer therapy [64].

The tumor- and metastasis-promoting function of LOX, in turn, has been linked in many studies to the lysyl oxidase enzyme activity, which is typically increased in cancer cells under hypoxic (low oxygen) conditions [65]. Inhibition of LOX activity by BAPN has been found to inhibit invasion and migration of tumor cells *in vitro* and to reduce metastasis formation *in vivo* [31, 65, 66], but no or only a marginal inhibition of cell proliferation by BAPN in standard 2D cultures has usually been observed [29, 58]. However, depending on the culture conditions, LOX activity has also been found to be needed for cell proliferation [53, 67, 68]. As a competitive inhibitor, the effects of BAPN may also be affected by the concentrations of the different substrates of LOX produced by different cell types. Here, we found that inhibition of LOX activity by BAPN had only a minimal, if any, effect on the proliferation of melanoma cells in 2D culture, but markedly inhibited the invasive growth of the cells in 3D Matrigel, particularly when co-cultured with fibroblasts. Notably, BAPN has also been reported to inhibit the activity of other LOX family members [69, 70], of which LOXL2 and LOXL3 were found to be expressed by both melanoma cells and fibroblasts, making the interpretation of the results complicated. To assess the specific roles played by the individual LOX family members, specific inhibitors, chemical compounds or antibody inhibitors, such as AB0023 for LOXL2 [70] would be needed. A couple of new, non-commercially available pharmacological inhibitors for LOX family members have recently been introduced [53, 71, 72], but the development of highly specific inhibitors is hampered by the fact that the crystal structures of the LOX family proteins are not yet resolved. Interestingly, our studies revealed that the depletion of LOX, and particularly that of LOXL2 by shRNAs inhibited both the proliferative capacity and the invasive growth capability of melanoma cells much more effectively than BAPN. Thus, the development of the enzyme activity-based inhibitors alone may not be sufficient, but we may have to invent various means to interfere with the expression or functions (including protein interactions) of the LOX family members to effectively combat cancer.

Besides the cancer cell intrinsic factors, tumor progression also depends on other cells. We have previously shown that the interaction between melanoma cells and fibroblasts, and their invasive growth in co-cultures in Matrigel is driven by TGFbeta [55], which also can induce the expression of all members of the LOX family [73]. In breast cancer cells, endogenous *LOX* and *LOXL2* expression has been shown to be upregulated when the cells were incubated with fibroblast-conditioned medium or plated on a fibroblast-conditioned collagen matrix [74]. Also reciprocally, LOXL2 secreted by cancer cells has been shown to activate fibroblasts and increase LOXL2 production in the fibroblasts [75]. Thus, co-culturing of melanoma cells and fibroblasts together may activate a feed-forward loop leading to increased expression of many LOX family proteins in both cell types. Hence, further studies are needed to clarify the specific roles of the different LOX proteins in the collaborating cells regulating melanoma cell behavior.

The function of LOX as a tumor suppressor versus promoter may thus depend, besides its processing and activity, on the cell type, stage of progression, cellular contexts, and the mode of cell migration/invasion. Interestingly, we found ODC-transformed fibroblasts to downregulate, in addition to *Lox*, the expression of many other ECM-related genes compared to parental NIH3T3 fibroblasts, including those encoding collagen I and fibronectin. As a consequence of this, the collagen I content and its crosslinking (and the deposition of fibrillar ECM in general) are reduced in the ODC-transformed cells compared to normal NIH3T3 cells (N1). We have previously shown that the N1 cells do not form tumors (or only form tiny tumors), while the ODC-transformed cells produce rapidly growing and locally invasive tumors in nude mice [22]. Hence, the formation of collagen I and its crosslinking in normal NIH3T3 cells is associated with tumor suppression. By contrast, we have found that melanoma tumors, like many other tumors, show upregulated expression of ECM-related genes, which may be expressed by both melanoma cells and stromal cells [76, 77 and this study]. Here, we found that also the expression of *LOX*, *LOXL1*, and *LOXL3* encoding proteins that crosslink ECM proteins (collagens and elastin) are increased in many melanoma cells and primary melanomas. Consequently, melanoma development and progression is associated with an increase in fibrillar network of ECM proteins (like collagen I, fibronectin, and periostin) around the melanoma cells [76]. Interestingly, fibronectin and periostin are also known to activate LOX [78, 79]. We have speculated that the tubular meshwork structures formed by these ECM proteins (together with matrix proteases) provide specific channels for melanoma tumor cells to invade and spread [76, 80]. In fact, many solid tumor cells have been suggested to use aligned crosslinked collagen bundles to migrate and invade [81]. However, a recent study in a pancreatic cancer model represents a caution that depletion of cancer-associated (αSMA^+^) fibroblasts and fibrosis may, surprisingly, accelerate pancreas cancer with reduced survival. Nonetheless, the expression of LOX appeared to remain high [82]. One explanation to this may be that during evolution (or artificial genetic manipulation) some cancer cells may gain independence of fibroblasts [76], or that other types of cancer-associated fibroblasts or other stromal cells compensate for the reduction in αSMA^+^ fibroblast functions.

Stromal or tumor cell-derived LOX expression has also been reported to play an important role in angiogenesis and tumor metastasis [83, 84]. Further, LOXL2 expression both in cancer cells and associated stromal fibroblasts has been shown to correlate with metastasis and poor survival in gastric cancer [85]. LOXL2 may also serve as a prognostic factor in melanoma as we found high LOXL2 mRNA expression in primary melanomas to be associated with formation of metastases and shorter survival of patients. These results remain interesting to be validated in a larger data set. Likewise, high LOXL2 mRNA expression has been found to associate with lymph node metastasis in esophageal squamous cell carcinoma [86] and with decreased overall survival in lung squamous cell carcinoma and lymph node-negative breast adenocarcinoma [87], and in colon cancer [88]. In addition, high LOXL2 protein expression has been found to associate with poorer overall survival in gastric cancer [89], laryngeal squamous cell carcinoma [87], and breast cancer [90].

In conclusion, in a search for genes involved in cell transformation, we found by microarray and RT-PCR analyses *Lox*, *Loxl1*, and *Loxl3* downregulation in ODC- and RAS-transformed mouse fibroblasts compared to normal fibroblasts, and upregulation of *Lox* in Odc-pLRT-TAM67 cells, upon induction of TAM67 expression. Induced re-expression of LOX in Odc-pLRT-LOX cells led to inhibition of cell growth and invasion in 3D Matrigel in an activity-independent manner. Our data thus suggest that the inactive pro-LOX acts as major tumor suppressor, in addition to the previously known LOX-PP. It will be interesting to see whether various possible means to increase the extracellular content of pro-LOX, e.g. by inhibiting the cleavage of pro-LOX with BMP1 inhibitors or with competitive pro-LOX-specific cleavage site peptides, would offer a viable strategy to inhibit tumor progression. Conversely, we found upregulation of *LOX* and other LOXL family members, especially *LOXL2* and *LOXL3*, in several human melanoma cell lines. *LOXL2* proved to be the most interesting among the LOX family members in melanomas, as it was significantly upregulated in clinical human melanomas compared to benign nevi, and it was associated with the formation of metastases and shorter survival of patients. We further show that the invasive growth of melanoma cells can be inhibited with ΒAPN, and even totally blocked by depletion of LOX and LOXL2 with shRNAs. Altogether, therapeutic targeting of the LOX family members, especially LOX and LOXL2, by promoting the tumor suppressive function of LOX and/or inhibiting the activity or expression of these enzymes, may offer viable ways to combat melanoma and other cancers.

## MATERIALS AND METHODS

### Patient samples

Fresh benign nevi from healthy volunteers and primary cutaneous melanomas (Breslow's thickness 0.6–27 mm) were obtained by surgical excision at Helsinki University Central Hospital, Helsinki, Finland, and processed as described previously [55, 77, 91]. Adjacent sections were subjected to pathological and immunohistochemical examinations. The protocols for taking the specimens were approved by the Ethics Committees of the Helsinki University Central Hospital. Further, all patients gave their informed consent.

### Cell culture

NIH3T3 mouse fibroblasts stably transfected with the human ODC cDNA (Odc; [21]) and the *HRAS*^Val12^ (c-Ha-*ras*^Val12^) oncogene (E4; [57]) were cultured in DMEM or α-MEM (both from Invitrogen/Thermo Fischer Scientific, Carlsbad, CA, USA) containing penicillin, streptomycin, and 5% fetal bovine serum (FBS; Invitrogen). NIH3T3 cells transfected only with the neomycin resistance gene (N1) were used as a control.

Odc cells stably transfected with a tetracycline-inducible expression system of the transactivation domain deletion mutant of c-Jun (pLRT-TAM67) [24] or lysyl oxidase (pLRT-LOX) were grown in α-MEM containing 50 μg/ml gentamicin (Invitrogen) and 5% TET system approved FBS (Clontech, Laboratories Inc., Mountain View, CA, USA).

Primary human melanocytes were isolated and cultured as previously described [76, 80, 92]. The melanoma cell lines WM793 (established from a vertical growth phase melanoma) and WM239 (established from a melanoma metastasis) were kindly provided by Dr. Meenhard Herlyn (Wistar Institute, Philadelphia, PA, USA). The vertical growth phase melanoma cell line WM115 and the metastatic cell line SK-MEL-28 were from ATCC-LCG Standards (Borås, Sweden). The metastatic melanoma cell lines SK-MEL-103 and SK-MEL-147 were kindly provided by Dr. Marisol Soengas (Spanish National Cancer Research Centre, Madrid, Spain), and MM170 cell line was from CellBank Australia (Westmead, Australia). WM793, WM115, WM239, MM170, and SK-MEL-28 melanoma cell lines were cultured in RPMI 1640 (Sigma-Aldrich, St. Louis, MO, USA) containing 10% FBS and antibiotics. SK-MEL-103, SK-MEL-147, and BLM were grown in DMEM containing 10% FBS and antibiotics [77]. Human primary embryonic skin fibroblasts (HES) and melanoma cells isolated from a primary tumor (EL29) were grown in RPMI 1640 containing 10% FBS and antibiotics. Human microvascular endothelial cells (HMVECs; Invitrogen) were grown in growth factor-supplemented Medium 131 (Invitrogen).

### Generation of NIH 3T3 cell lines carrying a tetracycline-inducible expression system of lysyl oxidase (pLRT-LOX)

Human LOX cDNA (HLO20 fragment; [93]) was first cloned into pBluescript and then inserted in XhoI/NotI-digested reverse tetracycline-regulated retroviral vector pLRT. The pLRT-*LOX* plasmid or empty vector were stably transfected into Odc cells by using Lipofectamine PLUS or Lipofectamine 2000 (Invitrogen). Selection of transfected cells was started after two days with 5 μg/ml blasticidin (Invitrogen) and continued for 1–2 weeks. After that, the stable transfectants were maintained in 1 μg/ml blasticidin. Several clones were first picked up by cylinder cloning and subcloned by single-cell cloning in 96-wells. The LOX expression was induced by 1 μg/ml doxycycline (Sigma-Aldrich) and the clones were analyzed by Northern blotting, reverse transcription-PCR (RT-PCR), and Western blotting. The best clones were selected for further studies.

### Short hairpin RNA lentiviral particle transduction of melanoma cells

WM793 cells were grown to 60% confluency and transduced with MISSION^®^ shRNA Lentiviral Transduction Particles (Sigma-Aldrich) targeting LOX (SHCLNV-NM_002317; clone TRCN0000045991), LOXL2 (SHCLNV-NM_002318; clones TRCN0000046195 and TRCN0000046197) or with MISSION^®^ Non-Mammalian shRNA Control Transduction Particles (SHC002V) in the presence of 8 μg/ml polybrene. Transductions were performed in duplicates in 96-well plates according to the manufacturer's instructions, except using 1 × 10^4^ cells for transfections.

### Fluorescent labeling of living cells

Living melanoma cells and fibroblasts were labeled with Fluorescent Celltracker Dyes before experiments as previously described [55]. Celltracker Green CMFDA was used for fibroblasts and Celltracker Red CMTPX (Molecular Probes/Invitrogen) for melanoma cells.

### Analysis of cell growth

To study the effect of LOX re-expression on cell growth, we used Odc cells carrying the tetracycline-inducible pLRT-LOX or empty vector. The cells were plated in triplicates in the absence or presence of doxycycline (1 μg/ml) and the total numbers of cells were counted with a Coulter particle counter (Beckman Coulter, Fullerton, CA, USA) after 3 and 5 days (medium changed after 3 days). To study the effect of ΒAPN on cell growth of Odc and WM793 cells, the cells were cultured in triplicates with 0, 250, or 500 μM BAPN and counted after 3 and 5 days. The medium and ΒAPN were changed after 3 days. In addition, the cell numbers of WM793 cells transduced with control shRNA, LOX shRNA, or two different LOXL2 shRNAs were counted 10 days after transduction with the shRNA lentiviral transduction particles.

### Analysis of apoptosis and cell death

Apoptosis and cell death were analyzed by using CellEvent™ Caspase-3/7 Green ReadyProbes™ Reagent and ReadyProbes™ Cell Viability Imaging kit, Blue/Red (Invitrogen by Thermo Fisher Scientific).

### Matrigel invasion assays

Growth factor-reduced Matrigel (BD Biosciences, Franklin Lakes, NJ) was used in 3D invasion assays essentially as previously described [24, 94]. Briefly, 1 × 10^4^ Odc-pLRT-LOX cells (pretreated or not with 1 μg/ml doxycycline for 1–3 days), 2 to 3 × 10^4^ melanoma cells, or 1 × 10^4^ melanoma cells mixed with 1 × 10^4^ HES cells (in co-culture assays; [55]) were seeded onto 24-well plates pre-coated with 300 μl of Matrigel diluted 1:3 in serum-free growth medium. Cells were allowed to adhere for 1 hour, after which a second 250-μl layer of Matrigel solution was added and allowed to polymerize. Finally, 500 μl of growth medium containing 5 to 10% serum was added to cover the Matrigel. The Matrigel layers and the growth medium were supplemented or not with 1 μg/ml doxycycline, and with or without the irreversible LOX inhibitor BAPN (0, 250, or 500 μM), [51]. The patterns of cell growth were monitored daily by microscopy and photographed after 1 to 4 days by using Olympus IX71 microscope and Olympus DP70 camera (both from Tokyo, Japan) [24, 55, 94, 95].

### RNA extraction

Polyadenylated mRNA for Northern blot analysis and cDNA LifeArrays was extracted by oligo(dT) cellulose chromatography [57]. Total RNA for RT-PCR and Affymetrix microarray analyses was isolated with the RNeasy kit (Qiagen, Crawley, UK) according to manufacturer's instructions.

### Northern blot analysis

LOX mRNA levels of the tetracycline-inducible LOX derivatives of Odc cells were confirmed by Northern blot analysis as previously described [95]. The single-stranded LOX antisense RNA probe was generated from the HLO20 sense fragment.

### Microarray analyses

Polyadenylated RNAs were extracted from the cells and analyzed with Mouse GEM2/Unigene1 cDNA LifeArrays (Incyte Genomics, Palo Alto, CA, USA), as previously described [95, 96]. Total RNA was extracted from the cells and hybridized to Affymetrix oligonucleotide MOE430 Set arrays, as previously described [95, 96]. Human benign nevi and primary melanomas were analyzed with HG-U133 Plus 2.0 Array (Affymetrix, Santa Clara, CA, USA), as previously described [91].

### Reverse transcription-PCR analysis

The expression levels of *LOX* and *LOXL1-4* were analyzed by RT-PCR essentially as previously described [95]. One μg of total RNA was used to synthesize cDNA, of which 1/10 (v/v) was subjected to PCR amplification. The mouse and human specific primers designed (from Proligo, Paris, France) and PCR variables are listed in Supplementary Tables 1 and 2, respectively. The primers for mouse *Loxl1* and *Loxl3* have previously been published in [79]. Glyceraldehyde-3-phosphate dehydrogenase (*Gapdh*) was used as a control with mouse cells and β-actin (*ACTB*) with human cells. The primers and PCR conditions for *Gapdh* and *ACTB* were the same as previously described [91, 95], except that the number of cycles for *Gapdh* was 20 and for *ACTB* 25. PCR products were separated on 2% agarose gels and visualized with Gel Star Nucleic Acid Gel Stain (Cambrex Bio Science Rockland, Rockland, ME, USA) under UV light. Gel Doc 2000 and Quantity One 4.2.3 software (Bio-Rad, Hercules, CA, USA) were used in gel documentation.

### Western blot analysis

Secreted proteins were first concentrated using Amicon Ultra Cel-10 centrifugal filters (MWCO 10.000; Millipore, Bedford, MA, USA) as previously described [94]. Cellular lysates or secreted proteins were resolved by 10% SDS-PAGE, and the proteins were electrophoretically transferred onto nitrocellulose membrane (Bio-Rad, Herwles, Hercules, CA, USA) and analyzed as previously [24]. Secreted and cellular protein bands of human melanoma cells and fibroblasts were quantified using Image Studio Lite (LI-COR Biotechnology, Lincoln, NE, USA). A rabbit polyclonal antibody recognizing the C-terminal domain of LOX (L4669; Sigma-Aldrich), a rabbit polyclonal LOX propeptide antibody to LOX-PP (NB110-41568; Novus Biologicals, Littleton, CO, USA), and a rabbit polyclonal antibody to LOXL2 (ab96233; Abcam, Cambridge, UK) were used to detect the respective proteins. Secreted protein samples were verified to contain comparable amounts of total proteins by silver staining. Mouse monoclonal antibodies to alpha-tubulin (DM1A; Abcam) and actin (JLA20; Merck, Millipore, Billerica, MA, USA) were used as loading controls for cellular proteins.

### Fluorimetric lysyl oxidase assay

Amplite™ Fluorimetric Lysyl Oxidase Assay Kit (ATT Bioquest^®^, Inc., Sunnyvale, CA, USA) was used to detect the activity of lysyl oxidase in the media of N1 and WM793 cells. After growing for two days in normal serum containing media the cells were washed and transferred to serum free media for 18 h. The conditioned media were collected, and secreted proteins were concentrated using Amicon Ultra Cel-10 centrifugal filters (MWCO 10.000; Millipore, Bedford, MA, USA), as previously described [94]. The lysyl oxidase activity in the concentrated media was measured in the presence of 0, 250, or 500 μM ΒAPN and 0.1% albumin. Recombinant LOXL2, rhLOXL2 (Bio-techne Ltd, Abingdon, UK), was used as a positive control.

### Statistical analysis

Data analyses were performed using a two-tailed Welch's *t*-test, where *p* < 0.05 was considered statistically significant. The Kaplan–Meier survival curves were compared with the use of log-rank test in SPSS 21.0 software (SPSS, Chicago, USA).

## SUPPLEMENTARY MATERIALS FIGURES AND TABLES



## Abbreviations

ODC: ornithine decarboxylase
LOX: lysyl oxidase
LOXL: lysyl oxidase-like
LOX-PP: lysyl oxidase propeptide
BAPN: B-aminopropionitrile
ECM: extracellular matrix

## References

[1] Meng Q, Xia Y (2011). c-Jun, at the crossroad of the signaling network. Protein Cell.

[2] Schreiber M, Kolbus A, Piu F, Szabowski A, Mohle-Steinlein U, Tian J, Karin M, Angel P, Wagner EF (1999). Control of cell cycle progression by c-Jun is p53 dependent. Genes Dev.

[3] Shaulian E, Karin M (2002). AP-1 as a regulator of cell life and death. Nat Cell Biol.

[4] Shaulian E, Schreiber M, Piu F, Beeche M, Wagner EF, Karin M (2000). The mammalian UV response: c-Jun induction is required for exit from p53-imposed growth arrest. Cell.

[5] Yang-Yen HF, Chiu R, Karin M (1990). Elevation of AP1 activity during F9 cell differentiation is due to increased c-jun transcription. New Biol.

[6] Lohman FP, Gibbs S, Fischer DF, Borgstein AM, van de Putte P, Backendorf C (1997). Involvement of c-JUN in the regulation of terminal differentiation genes in normal and malignant keratinocytes. Oncogene.

[7] Bos TJ, Margiotta P, Bush L, Wasilenko W (1999). Enhanced cell motility and invasion of chicken embryo fibroblasts in response to Jun over-expression. Int J Cancer.

[8] Jiao X, Katiyar S, Willmarth NE, Liu M, Ma X, Flomenberg N, Lisanti MP, Pestell RG (2010). c-Jun induces mammary epithelial cellular invasion and breast cancer stem cell expansion. J Biol Chem.

[9] Zhang Y, Pu X, Shi M, Chen L, Qian L, Song Y, Yuan G, Zhang H, Yu M, Hu M, Shen B, Guo N (2007). c-Jun, a crucial molecule in metastasis of breast cancer and potential target for biotherapy. Oncol Rep.

[10] Toft DJ, Rosenberg SB, Bergers G, Volpert O, Linzer DI (2001). Reactivation of proliferin gene expression is associated with increased angiogenesis in a cell culture model of fibrosarcoma tumor progression. Proc Natl Acad Sci U S A.

[11] Vleugel MM, Greijer AE, Bos R, van der Wall E, van Diest PJ (2006). c-Jun activation is associated with proliferation and angiogenesis in invasive breast cancer. Hum Pathol.

[12] Angel P, Karin M (1991). The role of Jun, Fos and the AP-1 complex in cell-proliferation and transformation. Biochim Biophys Acta.

[13] Vogt PK (2001). Jun, the oncoprotein. Oncogene.

[14] Tessari G, Ferrara C, Poletti A, Dubrovich A, Corsini A, Del Favero G, Naccarato R (1999). The expression of proto-oncogene c-jun in human pancreatic cancer. Anticancer Res.

[15] Gee JM, Barroso AF, Ellis IO, Robertson JF, Nicholson RI (2000). Biological and clinical associations of c-jun activation in human breast cancer. Int J Cancer.

[16] Mariani O, Brennetot C, Coindre JM, Gruel N, Ganem C, Delattre O, Stern MH, Aurias A (2007). JUN oncogene amplification and overexpression block adipocytic differentiation in highly aggressive sarcomas. Cancer Cell.

[17] Blau L, Knirsh R, Ben-Dror I, Oren S, Kuphal S, Hau P, Proescholdt M, Bosserhoff AK, Vardimon L (2012). Aberrant expression of c-Jun in glioblastoma by internal ribosome entry site (IRES)-mediated translational activation. Proc Natl Acad Sci U S A.

[18] Spangler B, Vardimon L, Bosserhoff AK, Kuphal S (2011). Post-transcriptional regulation controlled by E-cadherin is important for c-Jun activity in melanoma. Pigment Cell Melanoma Res.

[19] Lopez-Bergami P, Kim H, Dewing A, Goydos J, Aaronson S, Ronai Z (2010). c-Jun regulates phosphoinositide-dependent kinase 1 transcription: implication for Akt and protein kinase C activities and melanoma tumorigenesis. J Biol Chem.

[20] Nilsson JA, Keller UB, Baudino TA, Yang C, Norton S, Old JA, Nilsson LM, Neale G, Kramer DL, Porter CW, Cleveland JL (2005). Targeting ornithine decarboxylase in Myc-induced lymphomagenesis prevents tumor formation. Cancer Cell.

[21] Auvinen M, Paasinen A, Andersson LC, Hölttä E (1992). Ornithine decarboxylase activity is critical for cell transformation. Nature.

[22] Auvinen M, Laine A, Paasinen-Sohns A, Kangas A, Kangas L, Saksela O, Andersson LC, Hölttä E (1997). Human ornithine decarboxylase-overproducing NIH3T3 cells induce rapidly growing, highly vascularized tumors in nude mice. Cancer Res.

[23] Hölttä E, Paasinen-Sohns A, Povelainen M, Jarvinen K, Ravanko K, Kangas A (1998). Cell transformation by ornithine decarboxylase is associated with phosphorylation of the transactivation domain of c-Jun. Biochem Soc Trans.

[24] Kielosto M, Nummela P, Katainen R, Leaner V, Birrer MJ, Hölttä E (2004). Reversible regulation of the transformed phenotype of ornithine decarboxylase- and ras-overexpressing cells by dominant-negative mutants of c-Jun. Cancer Res.

[25] Kagan HM, Li W (2003). Lysyl oxidase: properties, specificity, and biological roles inside and outside of the cell. J Cell Biochem.

[26] Uzel MI, Scott IC, Babakhanlou-Chase H, Palamakumbura AH, Pappano WN, Hong HH, Greenspan DS, Trackman PC (2001). Multiple bone morphogenetic protein 1-related mammalian metalloproteinases process pro-lysyl oxidase at the correct physiological site and control lysyl oxidase activation in mouse embryo fibroblast cultures. J Biol Chem.

[27] Guo Y, Pischon N, Palamakumbura AH, Trackman PC (2007). Intracellular distribution of the lysyl oxidase propeptide in osteoblastic cells. Am J Physiol Cell Physiol.

[28] Payne SL, Fogelgren B, Hess AR, Seftor EA, Wiley EL, Fong SF, Csiszar K, Hendrix MJ, Kirschmann DA (2005). Lysyl oxidase regulates breast cancer cell migration and adhesion through a hydrogen peroxide-mediated mechanism. Cancer Res.

[29] Erler JT, Giaccia AJ (2006). Lysyl oxidase mediates hypoxic control of metastasis. Cancer Res.

[30] Lucero HA, Kagan HM (2006). Lysyl oxidase: an oxidative enzyme and effector of cell function. Cell Mol Life Sci.

[31] Yang X, Li S, Li W, Chen J, Xiao X, Wang Y, Yan G, Chen L (2013). Inactivation of lysyl oxidase by beta-aminopropionitrile inhibits hypoxia-induced invasion and migration of cervical cancer cells. Oncol Rep.

[32] El-Haibi CP, Bell GW, Zhang J, Collmann AY, Wood D, Scherber CM, Csizmadia E, Mariani O, Zhu C, Campagne A, Toner M, Bhatia SN, Irimia D (2012). Critical role for lysyl oxidase in mesenchymal stem cell-driven breast cancer malignancy. Proc Natl Acad Sci U S A.

[33] Kasashima H, Yashiro M, Kinoshita H, Fukuoka T, Morisaki T, Masuda G, Sakurai K, Kubo N, Ohira M, Hirakawa K (2016). Lysyl oxidase is associated with the epithelial-mesenchymal transition of gastric cancer cells in hypoxia. Gastric Cancer.

[34] Contente S, Kenyon K, Rimoldi D, Friedman RM (1990). Expression of gene rrg is associated with reversion of NIH 3T3 transformed by LTR-c-H-ras. Science.

[35] Kenyon K, Contente S, Trackman PC, Tang J, Kagan HM, Friedman RM (1991). Lysyl oxidase and rrg messenger RNA. Science.

[36] Kaneda A, Wakazono K, Tsukamoto T, Watanabe N, Yagi Y, Tatematsu M, Kaminishi M, Sugimura T, Ushijima T (2004). Lysyl oxidase is a tumor suppressor gene inactivated by methylation and loss of heterozygosity in human gastric cancers. Cancer Res.

[37] Bouez C, Reynaud C, Noblesse E, Thepot A, Gleyzal C, Kanitakis J, Perrier E, Damour O, Sommer P (2006). The lysyl oxidase LOX is absent in basal and squamous cell carcinomas and its knockdown induces an invading phenotype in a skin equivalent model. Clin Cancer Res.

[38] Xu X, Wang B, Xu Y (2013). Expression of lysyl oxidase in human osteosarcoma and its clinical significance: a tumor suppressive role of LOX in human osteosarcoma cells. Int J Oncol.

[39] Sakai M, Kato H, Sano A, Tanaka N, Inose T, Kimura H, Sohda M, Nakajima M, Kuwano H (2009). Expression of lysyl oxidase is correlated with lymph node metastasis and poor prognosis in esophageal squamous cell carcinoma. Ann Surg Oncol.

[40] Wilgus ML, Borczuk AC, Stoopler M, Ginsburg M, Gorenstein L, Sonett JR, Powell CA (2011). Lysyl oxidase: A lung adenocarcinoma biomarker of invasion and survival. Cancer.

[41] Baker AM, Cox TR, Bird D, Lang G, Murray GI, Sun XF, Southall SM, Wilson JR, Erler JT (2011). The role of lysyl oxidase in SRC-dependent proliferation and metastasis of colorectal cancer. J Natl Cancer Inst.

[42] Lee YS, Park Y, Kwon M, Roh JL, Choi SH, Nam SY, Kim SY (2017). Expression of Lysyl Oxidase Predictive of Distant Metastasis of Laryngeal Cancer. Otolaryngol Head Neck Surg.

[43] Molnar J, Fong KS, He QP, Hayashi K, Kim Y, Fong SF, Fogelgren B, Szauter KM, Mink M, Csiszar K (2003). Structural and functional diversity of lysyl oxidase and the LOX-like proteins. Biochim Biophys Acta.

[44] Zhan P, Shen XK, Qian Q, Zhu JP, Zhang Y, Xie HY, Xu CH, Hao KK, Hu W, Xia N, Lu GJ, Yu LK (2012). Down-regulation of lysyl oxidase-like 2 (LOXL2) is associated with disease progression in lung adenocarcinomas. Med Oncol.

[45] Gorogh T, Weise JB, Holtmeier C, Rudolph P, Hedderich J, Gottschlich S, Hoffmann M, Ambrosch P, Csiszar K (2007). Selective upregulation and amplification of the lysyl oxidase like-4 (LOXL4) gene in head and neck squamous cell carcinoma. J Pathol.

[46] Moreno-Bueno G, Salvador F, Martin A, Floristan A, Cuevas EP, Santos V, Montes A, Morales S, Castilla MA, Rojo-Sebastian A, Martinez A, Hardisson D, Csiszar K (2011). Lysyl oxidase-like 2 (LOXL2), a new regulator of cell polarity required for metastatic dissemination of basal-like breast carcinomas. EMBO Mol Med.

[47] Li RK, Zhao WY, Fang F, Zhuang C, Zhang XX, Yang XM, Jiang SH, Kong FZ, Tu L, Zhang WM, Yang SL, Cao H, Zhang ZG (2015). Lysyl oxidase-like 4 (LOXL4) promotes proliferation and metastasis of gastric cancer via FAK/Src pathway. J Cancer Res Clin Oncol.

[48] Nummela P, Lammi J, Soikkeli J, Saksela O, Laakkonen P, Hölttä E (2012). Transforming growth factor beta-induced (TGFBI) is an anti-adhesive protein regulating the invasive growth of melanoma cells. Am J Pathol.

[49] Min C, Kirsch KH, Zhao Y, Jeay S, Palamakumbura AH, Trackman PC, Sonenshein GE (2007). The tumor suppressor activity of the lysyl oxidase propeptide reverses the invasive phenotype of Her-2/neu-driven breast cancer. Cancer Res.

[50] Contente S, Yeh TJ, Friedman RM (2009). Tumor suppressive effect of lysyl oxidase proenzyme. Biochim Biophys Acta.

[51] Tang SS, Trackman PC, Kagan HM (1983). Reaction of aortic lysyl oxidase with beta-aminopropionitrile. J Biol Chem.

[52] Kagan HM, Reddy VB, Panchenko MV, Nagan N, Boak AM, Gacheru SN, Thomas KM (1995). Expression of lysyl oxidase from cDNA constructs in mammalian cells: the propeptide region is not essential to the folding and secretion of the functional enzyme. J Cell Biochem.

[53] Tang H, Leung L, Saturno G, Viros A, Smith D, Di Leva G, Morrison E, Niculescu-Duvaz D, Lopes F, Johnson L, Dhomen N, Springer C, Marais R (2017). Lysyl oxidase drives tumour progression by trapping EGF receptors at the cell surface. Nat Commun.

[54] Tang SS, Chichester CO, Kagan HM (1989). Comparative sensitivities of purified preparations of lysyl oxidase and other amine oxidases to active site-directed enzyme inhibitors. Connect Tissue Res.

[55] Yin M, Soikkeli J, Jahkola T, Virolainen S, Saksela O, Hölttä E (2012). TGF-beta signaling, activated stromal fibroblasts, and cysteine cathepsins B and L drive the invasive growth of human melanoma cells. Am J Pathol.

[56] Sistonen L, Keski-Oja J, Ulmanen I, Hölttä E, Wikgren BJ, Alitalo K (1987). Dose effects of transfected c-Ha-rasVal 12 oncogene in transformed cell clones. Exp Cell Res.

[57] Hölttä E, Sistonen L, Alitalo K (1988). The mechanisms of ornithine decarboxylase deregulation in c-Ha-ras oncogene-transformed NIH 3T3 cells. J Biol Chem.

[58] Palamakumbura AH, Jeay S, Guo Y, Pischon N, Sommer P, Sonenshein GE, Trackman PC (2004). The propeptide domain of lysyl oxidase induces phenotypic reversion of ras-transformed cells. J Biol Chem.

[59] Sato S, Trackman PC, Maki JM, Myllyharju J, Kirsch KH, Sonenshein GE (2011). The Ras signaling inhibitor LOX-PP interacts with Hsp70 and c-Raf to reduce Erk activation and transformed phenotype of breast cancer cells. Mol Cell Biol.

[60] Bais MV, Nugent MA, Stephens DN, Sume SS, Kirsch KH, Sonenshein GE, Trackman PC (2012). Recombinant lysyl oxidase propeptide protein inhibits growth and promotes apoptosis of pre-existing murine breast cancer xenografts. PLoS One.

[61] Agra N, Cidre F, Garcia-Garcia L, de la Parra J, Alonso J (2013). Lysyl oxidase is downregulated by the EWS/FLI1 oncoprotein and its propeptide domain displays tumor supressor activities in Ewing sarcoma cells. PLoS One.

[62] Vora SR, Palamakumbura AH, Mitsi M, Guo Y, Pischon N, Nugent MA, Trackman PC (2010). Lysyl oxidase propeptide inhibits FGF-2-induced signaling and proliferation of osteoblasts. J Biol Chem.

[63] Sanchez-Morgan N, Kirsch KH, Trackman PC, Sonenshein GE (2011). The lysyl oxidase propeptide interacts with the receptor-type protein tyrosine phosphatase kappa and inhibits beta-catenin transcriptional activity in lung cancer cells. Mol Cell Biol.

[64] Trackman PC (2016). Lysyl Oxidase Isoforms and Potential Therapeutic Opportunities for Fibrosis and Cancer. Expert Opin Ther Targets.

[65] Erler JT, Bennewith KL, Nicolau M, Dornhofer N, Kong C, Le QT, Chi JT, Jeffrey SS, Giaccia AJ (2006). Lysyl oxidase is essential for hypoxia-induced metastasis. Nature.

[66] da Silva R, Uno M, Marie SK, Oba-Shinjo SM (2015). LOX expression and functional analysis in astrocytomas and impact of IDH1 mutation. PLoS One.

[67] Pez F, Dayan F, Durivault J, Kaniewski B, Aimond G, Le Provost GS, Deux B, Clezardin P, Sommer P, Pouyssegur J, Reynaud C (2011). The HIF-1-inducible lysyl oxidase activates HIF-1 via the Akt pathway in a positive regulation loop and synergizes with HIF-1 in promoting tumor cell growth. Cancer Res.

[68] Cox TR, Erler JT (2013). Lysyl oxidase in colorectal cancer. Am J Physiol Gastrointest Liver Physiol.

[69] Jung ST, Kim MS, Seo JY, Kim HC, Kim Y (2003). Purification of enzymatically active human lysyl oxidase and lysyl oxidase-like protein from Escherichia coli inclusion bodies. Protein Expr Purif.

[70] Rodriguez HM, Vaysberg M, Mikels A, McCauley S, Velayo AC, Garcia C, Smith V (2010). Modulation of lysyl oxidase-like 2 enzymatic activity by an allosteric antibody inhibitor. J Biol Chem.

[71] Rowbottom MW, Bain G, Calderon I, Lasof T, Lonergan D, Lai A, Huang F, Darlington J, Prodanovich P, Santini AM, King CD, Goulet L, Shannon KE (2017). Identification of 4-(Aminomethyl)-6-(trifluoromethyl)-2-(phenoxy)pyridine Derivatives as Potent, Selective, and Orally Efficacious Inhibitors of the Copper-Dependent Amine Oxidase, Lysyl Oxidase-Like 2 (LOXL2). J Med Chem.

[72] Chang J, Lucas MC, Leonte LE, Garcia-Montolio M, Singh LB, Findlay AD, Deodhar M, Foot JS, Jarolimek W, Timpson P, Erler JT, Cox TR (2017). Pre-clinical evaluation of small molecule LOXL2 inhibitors in breast cancer. Oncotarget.

[73] Sethi A, Mao W, Wordinger RJ, Clark AF (2011). Transforming growth factor-beta induces extracellular matrix protein cross-linking lysyl oxidase (LOX) genes in human trabecular meshwork cells. Invest Ophthalmol Vis Sci.

[74] Kirschmann DA, Seftor EA, Fong SF, Nieva DR, Sullivan CM, Edwards EM, Sommer P, Csiszar K, Hendrix MJ (2002). A molecular role for lysyl oxidase in breast cancer invasion. Cancer Res.

[75] Barker HE, Bird D, Lang G, Erler JT (2013). Tumor-secreted LOXL2 activates fibroblasts through FAK signaling. Mol Cancer Res.

[76] Soikkeli J, Podlasz P, Yin M, Nummela P, Jahkola T, Virolainen S, Krogerus L, Heikkila P, von Smitten K, Saksela O, Hölttä E (2010). Metastatic outgrowth encompasses COL-I, FN1, and POSTN up-regulation and assembly to fibrillar networks regulating cell adhesion, migration, and growth. Am J Pathol.

[77] Eriksson J, Le Joncour V, Nummela P, Jahkola T, Virolainen S, Laakkonen P, Saksela O, Hölttä E (2016). Gene expression analyses of primary melanomas reveal CTHRC1 as an important player in melanoma progression. Oncotarget.

[78] Fogelgren B, Polgar N, Szauter KM, Ujfaludi Z, Laczko R, Fong KS, Csiszar K (2005). Cellular fibronectin binds to lysyl oxidase with high affinity and is critical for its proteolytic activation. J Biol Chem.

[79] Maruhashi T, Kii I, Saito M, Kudo A (2010). Interaction between periostin and BMP-1 promotes proteolytic activation of lysyl oxidase. J Biol Chem.

[80] Kaariainen E, Nummela P, Soikkeli J, Yin M, Lukk M, Jahkola T, Virolainen S, Ora A, Ukkonen E, Saksela O, Hölttä E (2006). Switch to an invasive growth phase in melanoma is associated with tenascin-C, fibronectin, and procollagen-I forming specific channel structures for invasion. J Pathol.

[81] Egeblad M, Rasch MG, Weaver VM (2010). Dynamic interplay between the collagen scaffold and tumor evolution. Curr Opin Cell Biol.

[82] Ozdemir BC, Pentcheva-Hoang T, Carstens JL, Zheng X, Wu CC, Simpson TR, Laklai H, Sugimoto H, Kahlert C, Novitskiy SV, De Jesus-Acosta A, Sharma P, Heidari P (2014). Depletion of carcinoma-associated fibroblasts and fibrosis induces immunosuppression and accelerates pancreas cancer with reduced survival. Cancer Cell.

[83] Osawa T, Ohga N, Akiyama K, Hida Y, Kitayama K, Kawamoto T, Yamamoto K, Maishi N, Kondoh M, Onodera Y, Fujie M, Shinohara N, Nonomura K (2013). Lysyl oxidase secreted by tumour endothelial cells promotes angiogenesis and metastasis. Br J Cancer.

[84] Pickup MW, Laklai H, Acerbi I, Owens P, Gorska AE, Chytil A, Aakre M, Weaver VM, Moses HL (2013). Stromally derived lysyl oxidase promotes metastasis of transforming growth factor-beta-deficient mouse mammary carcinomas. Cancer Res.

[85] Kasashima H, Yashiro M, Kinoshita H, Fukuoka T, Morisaki T, Masuda G, Sakurai K, Kubo N, Ohira M, Hirakawa K (2014). Lysyl oxidase-like 2 (LOXL2) from stromal fibroblasts stimulates the progression of gastric cancer. Cancer Lett.

[86] Li TY, Xu LY, Wu ZY, Liao LD, Shen JH, Xu XE, Du ZP, Zhao Q, Li EM (2012). Reduced nuclear and ectopic cytoplasmic expression of lysyl oxidase-like 2 is associated with lymph node metastasis and poor prognosis in esophageal squamous cell carcinoma. Hum Pathol.

[87] Peinado H, Moreno-Bueno G, Hardisson D, Pérez-Gómez E, Santos V, Mendiola M, de Diego JI, Nistal M, Quintanilla M, Portillo F, Cano A (2008). Lysyl oxidase-like 2 as a new poor prognosis marker of squamous cell carcinomas. Cancer Res.

[88] Torres S, Garcia-Palmero I, Herrera M, Bartolomé RA, Peña C, Fernandez-Aceñero MJ, Padilla G, Peláez-García A, Lopez-Lucendo M, Rodriguez-Merlo R, García de Herreros A, Bonilla F, Casal JI (2015). LOXL2 Is Highly Expressed in Cancer-Associated Fibroblasts and Associates to Poor Colon Cancer Survival. Clin Cancer Res.

[89] Peng L, Ran YL, Hu H, Yu L, Liu Q, Zhou Z, Sun YM, Sun LC, Pan J, Sun LX, Zhao P, Yang ZH (2009). Secreted LOXL2 is a novel therapeutic target that promotes gastric cancer metastasis via the Src/FAK pathway. Carcinogenesis.

[90] Ahn SG, Dong SM, Oshima A, Kim WH, Lee HM, Lee SA, Kwon SH, Lee JH, Lee JM, Jeong J, Lee HD, Green JE (2013). LOXL2 expression is associated with invasiveness and negatively influences survival in breast cancer patients. Breast Cancer Res Treat.

[91] Soikkeli J, Lukk M, Nummela P, Virolainen S, Jahkola T, Katainen R, Harju L, Ukkonen E, Saksela O, Hölttä E (2007). Systematic search for the best gene expression markers for melanoma micrometastasis detection. J Pathol.

[92] Alanko T, Rosenberg M, Saksela O (1999). FGF expression allows nevus cells to survive in three-dimensional collagen gel under conditions that induce apoptosis in normal human melanocytes. J Invest Dermatol.

[93] Hamalainen ER, Jones TA, Sheer D, Taskinen K, Pihlajaniemi T, Kivirikko KI (1991). Molecular cloning of human lysyl oxidase and assignment of the gene to chromosome 5q23.3–31.2. Genomics.

[94] Ravanko K, Jarvinen K, Helin J, Kalkkinen N, Hölttä E (2004). Cysteine cathepsins are central contributors of invasion by cultured adenosylmethionine decarboxylase-transformed rodent fibroblasts. Cancer Res.

[95] Nummela P, Yin M, Kielosto M, Leaner V, Birrer MJ, Hölttä E (2006). Thymosin beta4 is a determinant of the transformed phenotype and invasiveness of S-adenosylmethionine decarboxylase-transfected fibroblasts. Cancer Res.

[96] Kielosto M, Nummela P, Jarvinen K, Yin M, Hölttä E (2009). Identification of integrins alpha6 and beta7 as c-Jun- and transformation-relevant genes in highly invasive fibrosarcoma cells. Int J Cancer.

