# Supplementary material for: Divergent roles of lysyl oxidase family members in ornithine decarboxylase- and RAS-transformed mouse fibroblasts and human melanoma cells
Source: Oncotarget. 2018 Dec 28;9(102):37733–52. doi: 10.18632/oncotarget.26508 (PMC6340875; doi:10.18632/oncotarget.26508)
Supplement: Supplementary file 1 [file oncotarget-09-37733-s001.pdf]

Divergent roles of lysyl oxidase family members in ornithine decarboxylase- and RAS-transformed mouse fibroblasts and human melanoma cells

SUPPLEMENTARY MATERIALS

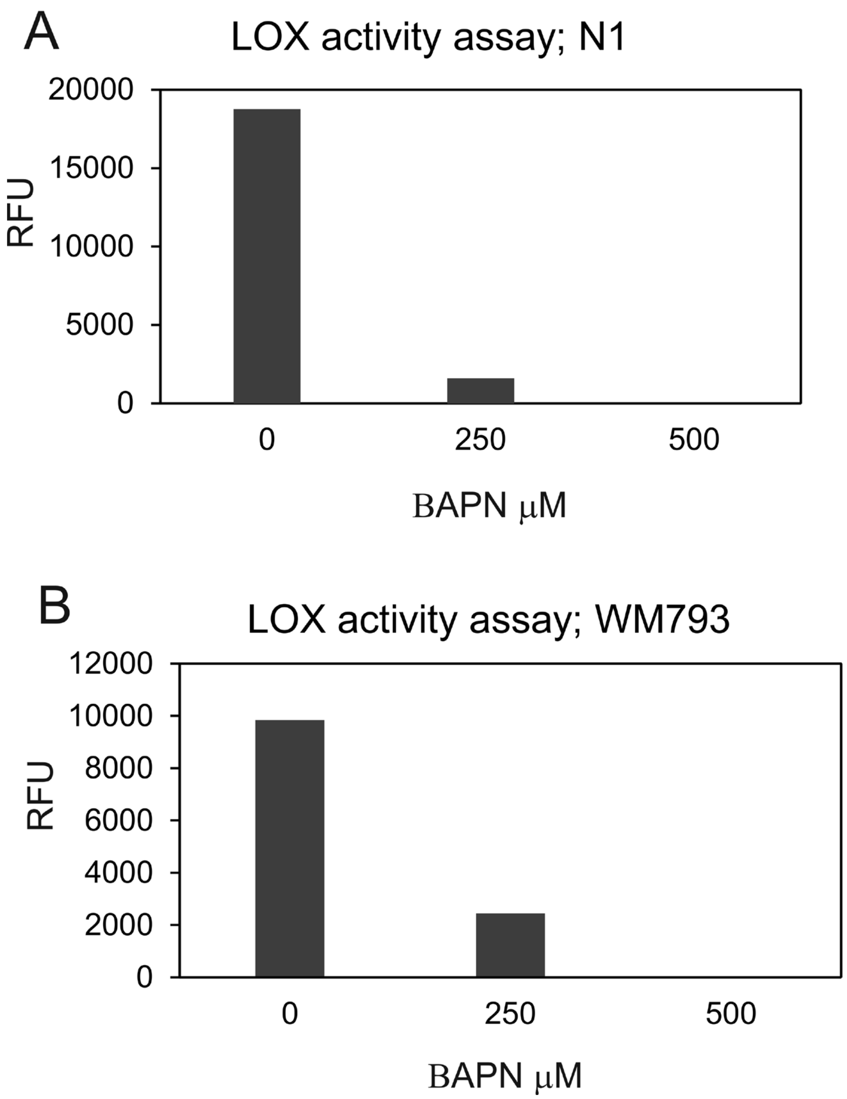

Supplementary Figure 1: Inhibition of the LOX activity in normal N1 fibroblasts and WM793 cells by β-aminopropionitrile (BAPN). (**A** and **B**) LOX activity in the conditioned media of N1 cells (A) and WM793 cells (B) cultured in the presence of 0, 250, or 500 μM BAPN as described in the Materials and methods. RFU = relative fluorescence units.

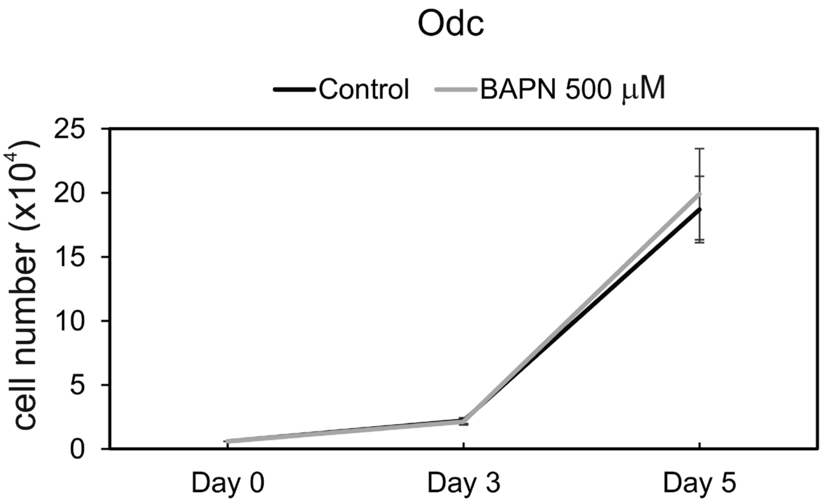

Supplementary Figure 2: Effect of BAPN on the proliferation of Odc cells. Growth curves of Odc cells cultured in the absence or presence of 500 μM BAPN and calculated after 3 and 5 days.

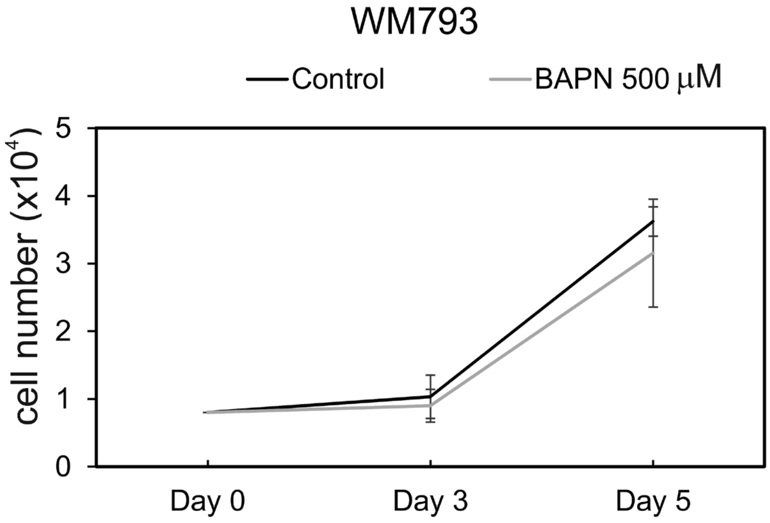

Supplementary Figure 3: Effect of BAPN on the proliferation of WM793 cells in sparse cultures. Growth curves of sparsely populated WM793 cells (8×10^3 cells) seeded on a 6-well plate in the absence or presence of 500 μM BAPN.

Supplementary Table 1: Mouse-specific PCR primers

Gene name	Orientation	Sequence	Annealing (°C)	Cycles*
Lox	fw	5′-TGGCACAGCTGTCACCAACATT-3′	61	25
	rv	5′-ACAGAAGCTTGCTTTGTGGCCT-3′		
Loxl1	fw	5'-GCATCCACATACGTGCAGAG-3'	62	25
	rv	5'-GGTCGTAGTGGCTGAACTCG-3'		
Loxl2	fw	5'-GTGCCAACTTTGGAGAACAAGGCA-3'	61	30
	rv	5'-TTGTACATCCAGATGCGGTAGCCA-3'		
Loxl3	fw	5'-AGAACATCACAGCTGAGGAC-3'	64	29
	rv	5'-GCTCATCACCACCTCAGTTAC-3'		
Loxl4	fw	5'-AAGTGGTGATGAGTGGAGTTCGCT-3'	62	30
	rv	5'-TTTCCTCGTGAGCACAGTACAGCA-3'		

*The numbers of cycles were optimized to be in the linear range.
Abbreviations: fw = forward, rv = reverse.

Supplementary Table 2: Human-specific PCR primers

Gene name	Orientation	Sequence	Annealing (°C)	Cycles*
LOX	fw	5′-ATGATCACAGGGTGCTGCTCAGAT-3′	61	20
	rv	5′-GTGTGCAGTACATGCAAATCGCCT-3′		
LOXL1	fw	5'-ATCCACTTATGTGCAGAGAGCCCA-3'	62	24
	rv	5'-AGTCGATGTCCGCATTGTAGGTGT-3'		
LOXL2	fw	5'-AGCTTCTGCTTGGAGGACACAGAA-3'	60	22
	rv	5'-GCGGCTCCTGCATTTCATGATGTT-3'		
LOXL3	fw	5'-TGTTGTACTGTGCTGCGGAAGAGA-3'	60	22
	rv	5'-GAAGGCATCACCAATGTGGCAGTT-3'		
LOXL4	fw	5'-GCATGACATTGATTGCCAGTGGGT-3'	64	20
	rv	5'-ATGAGGTTGTTCCTGAGACGCTGT-3'		

*The numbers of cycles were optimized to be in the linear range.
Abbreviations: fw = forward, rv = reverse.
